# Real-world disproportionality analysis of cardiac failure associated with novel antineoplastic agents in breast cancer: a pharmacovigilance study

**DOI:** 10.3389/fimmu.2025.1680909

**Published:** 2025-09-29

**Authors:** Hui Li, YingJia Wu, Qin Guo, ChengShan Guo, FangJie Liu, LanQing Huo

**Affiliations:** ^1^ Department of Rheumatology and Immunology, The People’s Hospital of Baoan Shenzhen, The Second Affiliated Hospital of Shenzhen University, Shenzhen, China; ^2^ Department of Radiation Oncology, State Key Laboratory of Oncology in South China, Guangdong Provincial Clinical Research Center for cancer, Sun Yat−sen University Cancer Center, Guangzhou, China

**Keywords:** breast cancer, cardiac failure, pharmacovigilance, disproportionality analysis, novel antineoplastic agent

## Abstract

**Background:**

Some antineoplastic agents have been implicated in cardiac failure (CF), but large-scale real-world data remain limited. This study aimed to assess disproportional reporting signals for CF associated with novel antineoplastic agents used in breast cancer treatment.

**Methods:**

A disproportionality analysis was conducted using individual case safety reports from the FDA Adverse Event Reporting System (FAERS, Jan 2004–Mar 2025), Canada Vigilance Adverse Reaction Database (CANADA, Jan 2004–Dec 2024), and Japanese Adverse Drug Event Report (JADER, Jan 2004–Oct 2024). Reports involving breast cancer patients treated with FDA-approved targeted agents were included. Reporting odds ratios (RORs), time-to-onset (TTO), and death proportion were evaluated by drug and class.

**Results:**

A total of 8,565 CF cases were identified across the three databases. Trastuzumab exhibited consistently strong signals (FAERS: ROR 2.94; CANADA: 6.15; JADER: 7.05), with pertuzumab and everolimus showing variable significance. CDK4/6 inhibitors and immune checkpoint inhibitors (ICIs) demonstrated low or inverse RORs. Median TTO was longest for monoclonal antibodies, and shorter for ADCs and ICIs. Everolimus (20.1%), sacituzumab govitecan (22.6%), and atezolizumab (23.5%) showed the highest death proportions, while trastuzumab had a lower proportion (8.75%). HER2-targeted agents had higher reporting signal of CF than traditional chemotherapies.

**Conclusion:**

This study underscores drug-specific differences in CF risk among breast cancer therapies and highlights the importance of individualized cardiovascular risk assessment during treatment planning and monitoring.

## 1 Introduction

Breast cancer is the most commonly diagnosed cancer among women worldwide and remains a leading cause of cancer-related morbidity and mortality. According to GLOBOCAN data, over 2.3 million new cases of breast cancer were diagnosed globally in 2022, with a steady increase in incidence observed over the past decade (1). Advances in molecular subtyping and precision medicine have transformed the treatment landscape of breast cancer, particularly through the development and approval of novel antineoplastic agents. Among these, HER2-targeted monoclonal antibodies (mAbs) (2), antibody-drug conjugates (ADCs) (3), cyclin-dependent kinase 4/6 (CDK4/6) inhibitors (4), immune checkpoint inhibitors (ICIs) (5), poly adenosine diphosphate-ribose polymerase (PARP) inhibitors (6), and phosphatidylinositol 3-kinase (PI3K)/protein kinase B (AKT)/mammalian target of rapamycin (mTOR) inhibitors (7), tyrosine kinase inhibitors (TKIs) (8) have significantly improved survival outcomes in various subtypes of breast cancer. As breast cancer is increasingly treated as a chronic disease, especially in patients with hormone receptor-positive or HER2-positive subtypes, the cumulative exposure to these agents raises concerns about long-term toxicity. Serious adverse events, such as cardiotoxicity and interstitial lung disease, may significantly impact quality of life and disrupt ongoing care. This growing awareness has prompted increased attention toward pharmacovigilance efforts and real-world safety data to inform clinical decision-making and risk-benefit assessments.

Cardiotoxicity, including cardiac failure (CF), has emerged as a critical concern in breast cancer survivorship (9). Breast cancer patients may be particularly susceptible to cardiotoxicity due to several factors: frequent exposure to multimodal treatment regimens with overlapping cardiac risks, the increasing prevalence of baseline cardiovascular comorbidities, and the prolonged use of targeted therapies in both early and metastatic settings (10). In the context of cancer therapy, drug-induced CF may manifest as asymptomatic reductions in left ventricular ejection fraction (LVEF), symptomatic congestive CF, or even fatal cardiac events. Trastuzumab, a HER2-targeted mAb, is one of the most well-documented agents associated with cardiotoxicity, leading to the incorporation of routine cardiac monitoring in treatment protocols (11). However, emerging agents, such as trastuzumab deruxtecan, CDK4/6 inhibitors, and PARP inhibitors have less well-defined cardiac safety profiles. A systematic review including 15 studies reported an overall incidence of decreased LVEF at 1.95% among patients receiving trastuzumab deruxtecan (12). Similarly, CDK4/6 inhibitors have been associated with QT prolongation, hypertension, and, in rare cases, CF (13, 14). Despite these concerns, cardiac toxicity remains an underreported and underrecognized adverse effect in many clinical trials. This is partly due to stringent patient selection (e.g., exclusion of patients with pre-existing cardiovascular disease) and relatively short follow-up durations, which may underestimate the true incidence of delayed-onset or cumulative cardiotoxicity. Furthermore, traditional clinical trials often lack the statistical power to detect rare but serious adverse events such as CF.

Given the inherent limitations of randomized controlled trials (RCTs), real-world evidence derived from post-marketing surveillance has become an increasingly critical component in characterizing the safety profile of novel anticancer therapies. Spontaneous reporting systems, such as the Food and Drug Administration’s (FDA) Adverse Event Reporting System (FAERS), the Canada Vigilance Adverse Reaction Online Database (CANADA), and Japanese Adverse Drug Event Report database (JADER), provide large-scale, real-world datasets for pharmacovigilance research. These systems allow for the systematic detection of rare but serious adverse events, including drug-induced CF, through disproportionality analysis, a statistical approach that compares the observed reporting frequency of specific drug-event combinations against expected frequencies within the database. Compared with RCTs, those datasets reflect a broader and more heterogeneous patient population. Consequently, they can reveal safety signals that may be underrecognized or undetectable in controlled settings. Moreover, differences in healthcare systems, prescribing practices, and pharmacovigilance infrastructure across countries enable complementary insights when multiple international databases are analyzed in parallel.

Previous studies investigating cardiotoxicity in breast cancer have predominantly focused on individual agents or specific drug classes, with limited comparative assessments across different novel therapeutic categories (15–19). In contrast, the present study represents the first large-scale real-world pharmacovigilance analysis to systematically evaluate CF signals associated with a broad range of novel antineoplastic agents approved for breast cancer. By leveraging three major large adverse reaction databases, we provide a comprehensive and comparative assessment of CF risk across multiple drug classes. These findings offer new insights into the cardiac safety profiles of contemporary breast cancer therapies and underscore the importance of cross-drug and cross-class signal detection in the context of long-term survivorship.

The present study aimed to evaluate the association between novel antineoplastic agents and the reporting of CF in breast cancer using disproportionality analyses across three major pharmacovigilance databases. By integrating data from diverse geographic regions and healthcare settings, this analysis seeks to enhance the detection and validation of cardiotoxicity signals. The findings may inform clinical decision-making, guide post-marketing surveillance priorities, and contribute to a more nuanced understanding of cardiovascular risks in breast cancer therapeutics.

## 2 Methods

### 2.1 Study design and data source

**Figure 1:**
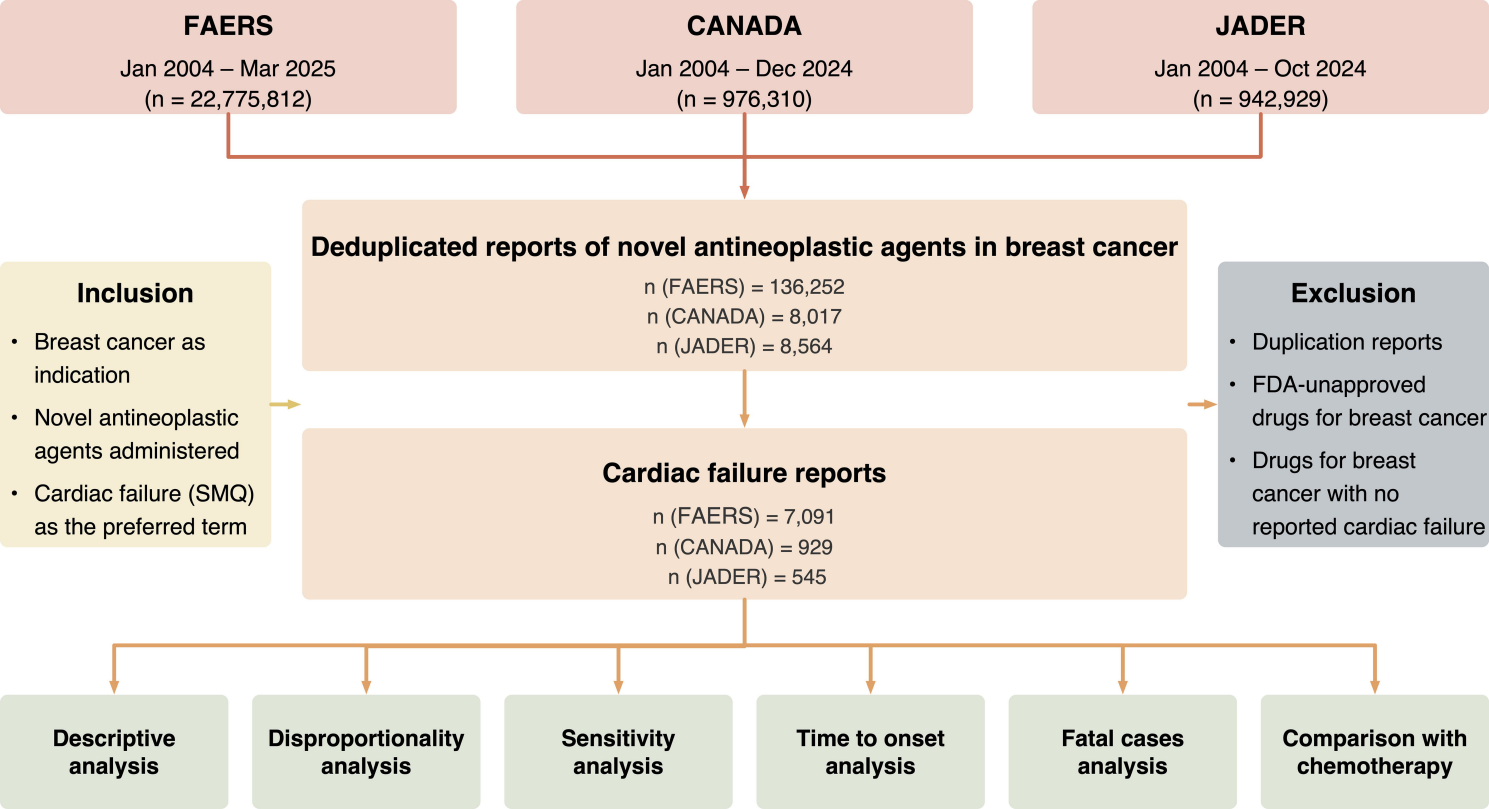
Flow chart of the study. FAERS, Food and Drug Administration’s Adverse Event Reporting System; CANADA, Canada Vigilance Adverse Reaction Online Database; JADER, Japanese Adverse Drug Event Report; SMQ, standardized medical dictionary for regulatory activities query.

**Figure 1** provides an overview of the disproportionality analysis, which was based on individual case safety reports (ICSRs) from three publicly available pharmacovigilance databases: FAERS, CANADA, and JADER. FAERS, administered by the US FDA, provided data from January 2004 to March 2025, including demographic information, drug exposure details, adverse event reports, outcomes, sources of reports, treatment periods, and indications for drug use. Reports from CANADA, managed by Health Canada, spanned January 2004 to December 2024 and encompassed standardized fields covering patient demographics, drug administration history, Medical Dictionary for Regulatory Activities (MedDRA)-coded adverse events, clinical outcomes, reporter information, and administrative data. JADER, maintained by the Pharmaceuticals and Medical Devices Agency of Japan, contributed data from January 2004 to October 2024, organized into distinct datasets covering demographics, drugs, adverse events, and primary diseases. All adverse events were coded using preferred terms (PTs) from MedDRA (version 27.1). Due to the anonymized and publicly accessible nature of the databases, ethical approval and patient consent were not required.

### 2.2 Procedures

Adverse drug reaction reports were screened to identify cases associated with FDA-approved novel antineoplastic agents specifically indicated for breast cancer treatment. Included medications comprised TKIs (lapatinib, neratinib, tucatinib), mAbs (trastuzumab, pertuzumab), ADCs (trastuzumab emtansine, trastuzumab deruxtecan, sacituzumab govitecan), ICIs (pembrolizumab, atezolizumab), CDK4/6 inhibitors (palbociclib, ribociclib, abemaciclib), PARP inhibitors (olaparib, niraparib, talazoparib), and PI3K/AKT/mTOR inhibitors (alpelisib, everolimus, capivasertib). For FAERS, CANADA, and JADER, drug names (generic and brand) were standardized using MeSH terms. Additionally, for JADER, nomenclature was further harmonized with the Japanese Accepted Names for Pharmaceuticals (JAN) database (https://jpdb.nihs.go.jp/jan). A comprehensive list of all drugs analyzed, including generic names, sources (MeSH or JAN), and search terms, is provided in **Supplementary Table S1**. CF was identified via the Standardized MedDRA Query (SMQ) for “cardiac failure”, with relevant PTs detailed in **Supplementary Table S2**. Reports were eligible if breast cancer was explicitly stated as the indication, administration of at least one of the specified agents was confirmed, and CF was recorded per SMQ definitions. Exclusion criteria encompassed duplicate reports, cases involving medications not approved for breast cancer, or instances lacking documented CF.

Data deduplication was performed in accordance with the FDA’s official documentation using R software. Specifically, we extracted the PRIMARYID, CASEID, and FDA_DT variables from the DEMO table and applied the following rules: (1) Records were sorted by CASEID, FDA_DT, and PRIMARYID. (2) For reports with the same CASEID, only the record with the most recent FDA_DT was retained. (3) If both CASEID and FDA_DT were identical, the record with the largest PRIMARYID was kept. After deduplication, each CASEID and PRIMARYID pair was unique, ensuring that, for the same patient, only the latest submitted report was preserved. Additionally, analysis inclusion required the study drug to be designated as the primary suspect medication (FAERS) or suspected medication (CANADA and JADER). All methodological processes conformed to the READUS-PV guideline to ensure transparency and reproducibility (20).

### 2.3 Statistical analysis

The main analysis involved calculating reporting odds ratios (RORs) with 95% confidence intervals (CIs) to evaluate disproportionality signals for CF related to each novel antineoplastic agent. A statistically significant disproportionality signal was defined when the lower bound of the 95% CI exceeded one and at least three cases were reported (21). To assess the robustness of these results, the Bayesian confidence propagation neural network (BCPNN) method was additionally applied. This method estimates the information component (IC) and its 95% credibility interval, with a signal considered present when the lower bound IC_025_ is greater than zero. The formulas for calculating RORs, CIs, and IC values are provided in the **Supplementary Materials**. Following signal detection, baseline demographic and clinical features of cases were summarized. The time from treatment initiation to CF onset (time-to-onset, TTO) was analyzed using median, quartiles, and Weibull distribution parameters, namely scale (alpha) and shape (beta). The Weibull shape parameter (beta) was interpreted as follows: values less than 1 indicated a decreasing risk over time, values close to 1 indicated a constant risk, and values greater than 1 indicated an increasing risk. Kaplan-Meier survival analysis and log-rank tests compared TTO among drug classes. Death proportions from CF associated with each agent were computed and compared using Pearson chi-square or Fisher’s exact test with Benjamini-Hochberg adjusted *P*-values below 0.05 indicating significance. Additionally, novel antineoplastic agents were compared to traditional chemotherapy drugs through comparative disproportionality analyses, calculating odds ratios and 95% CIs. Volcano plots illustrated these differences by visually presenting ROR values and Fisher’s exact test adjusted *P*-values.

Sensitivity analyses assessed robustness and potential confounding influences. External positive (rosiglitazone, known CF risk) and negative (tamoxifen, no known CF association) control drugs were included to validate analytic sensitivity. Multivariate logistic regression models adjusted for age, weight (excluding CANADA due to data limitations), reporter type, reporting region (excluding CANADA and JADER), co-administered medications, and potentially cardiotoxic concomitant drugs among the top 50 co-administered agents, excluding cases missing key covariate data. Additional sensitivity tests included analyses restricted to narrow SMQ-defined CF terms and disproportionality analyses limited solely to reports submitted by healthcare professionals. All analyses were conducted using R software (version 4.5.0) within the RStudio environment.

## 3 Results

### 3.1 Descriptive analysis

A total of 7,091, 929, and 545 breast cancer patients with 8,124, 1,049, and 586 reported CF events were identified in the FAERS, CANADA, and JADER databases, respectively. Most patients were female (FAERS 98.91%; CANADA 99.56%; JADER 99.81%), consistent with breast cancer epidemiology. The median age at the time of adverse event reporting was 62 years (interquartile range [IQR] 52–71) in FAERS and 57 years (IQR 47–65) in CANADA database. The median weight was 67kg (IQR 56–80) in FAERS and 71kg (IQR 61–81) in CANADA. Among cases with outcome data, the fatality proportion was 18.26% in FAERS, 14.57% in CANADA, and 6.28% in JADER. Hospitalization was the most common outcome in FAERS (n=1790; 31.38%) (**Table 1**).

**Table 1:** Clinical characteristics of patients with breast cancer associated CF in FAERS, CANADA, and JADER.

Characteristics	FAERS n. (%)	CANADA n. (%)	JADER n. (%)
Number of patients	7091	929	545
Gender			
Data available	6411	904	539
Female	6341 (98.91%)	900 (99.56%)	538 (99.81%)
Male	70 (1.09%)	4 (0.44%)	1 (0.19%)
Age at reporting (years)			
Data available	4874	612	480
<30	90 (1.85%)	63 (10.29%)	1 (0.21%)
30–39	213 (4.37%)	31 (5.07%)	8 (1.67%)
40–49	644 (13.21%)	86 (14.05%)	59 (12.29%)
50–59	1168 (23.96%)	199 (32.52%)	127 (26.46%)
60–69	1354 (27.78%)	139 (22.71%)	143 (29.79%)
70–79	1058 (21.71%)	82 (13.40%)	95 (19.79%)
≥80	347 (7.12%)	12 (1.96%)	47 (9.79%)
Median (IQR)	62 (52–71)	57 (47–65)	NA
Weight (kg)			
Data available	2271	132	198
<40	32 (1.41%)	1 (0.76%)	31 (16.66%)
40–49	211 (9.29%)	10 (7.58%)	62 (31.31%)
50–59	480 (21.14%)	17 (12.88%)	62 (31.31%)
60–69	535 (23.56%)	23 (17.42%)	36 (18.18%)
70–79	434 (19.10%)	42 (31.82%)	4 (2.02%)
80–89	280 (12.33%)	20 (15.15%)	NA
≥80	NA	NA	3 (1.52%)
≥90	299 (13.17%)	19 (14.39%)	NA
Median (IQR)	67 (56–80)	71 (61–81)	NA
Reported countries (Top 10)			
United States	2549 (35.95%)	NA	NA
Japan	487 (6.87%)	NA	NA
Canada	435 (6.13%)	NA	NA
Germany	381 (5.37%)	NA	NA
France	305 (4.30%)	NA	NA
Italy	253 (3.57%)	NA	NA
United Kingdom	231 (3.26%)	NA	NA
India	188 (2.65%)	NA	NA
China	166 (2.34%)	NA	NA
Argentina	165 (2.33%)	NA	NA
Outcomes			
Data available	5705	453	462
Death	1042 (18.26%)	66 (14.57%)	29 (6.28%)
Life-threatening	266 (4.66%)	NA	NA
Hospitalization	1790 (31.38%)	NA	NA
Disability	66 (1.16%)	NA	NA
Other serious	2541 (44.54%)	NA	NA
Recovery	NA	52 (11.48%)	181 (39.18%)
Remission	NA	56 (12.36%)	137 (29.65%)
Unrecovered	NA	275 (60.71%)	112 (24.24%)
With aftereffect	NA	4 (0.88%)	3 (0.65%)

Some characteristics are only reported in one of the databases (FAERS, CANADA, or JADER), resulting in NA for these categories in the other database. CF, cardiac failure; FAERS, Food and Drug Administration’s Adverse Event Reporting System; CANADA, Canada Vigilance Adverse Reaction Online Database; JADER, Japanese Adverse Drug Event Report; IQR, interquartile range; NA, not applicable.

### 3.2 Disproportionality analysis

**Figure 2:**
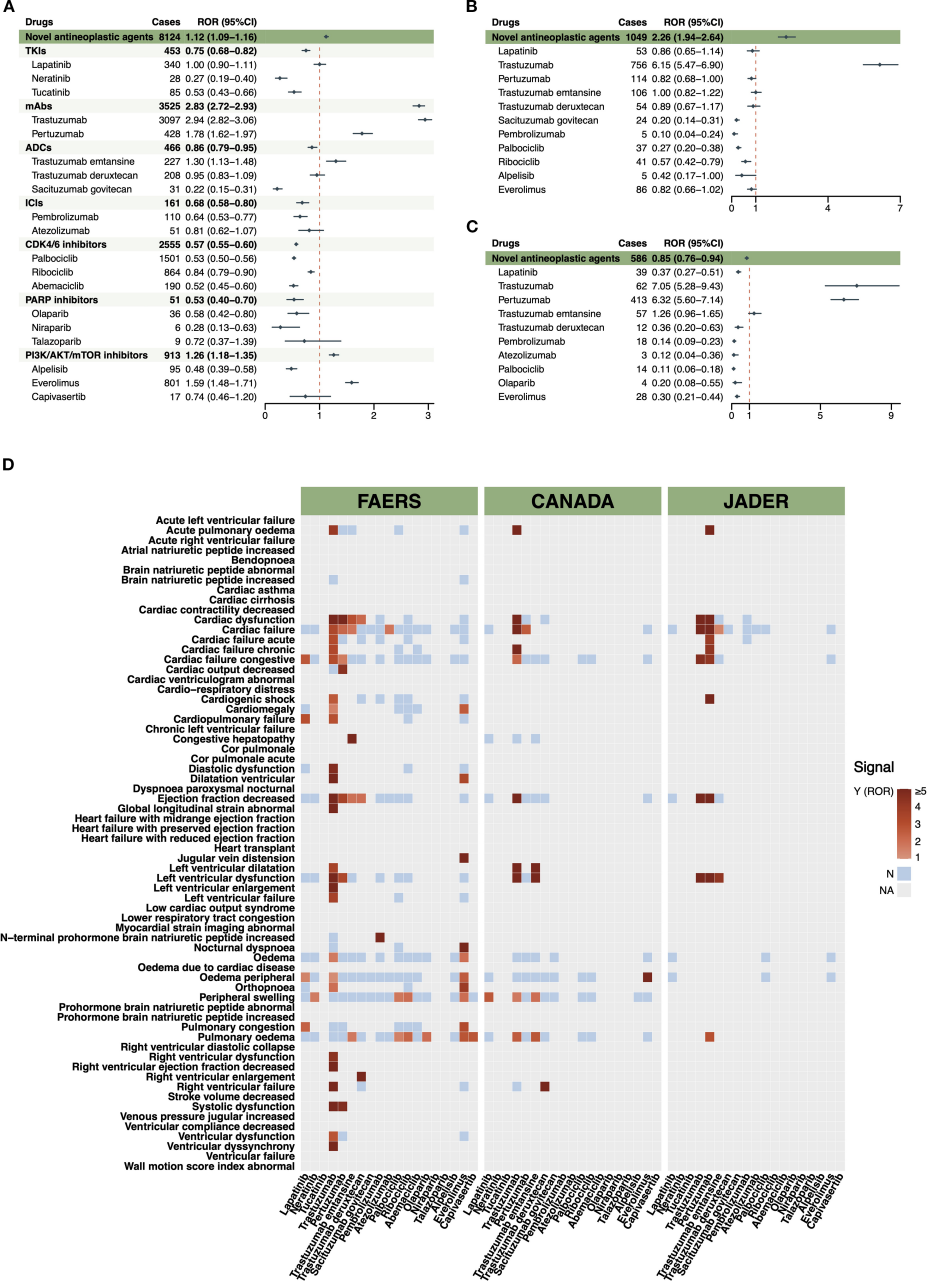
Disproportionality signals of cardiac failure associated with novel antineoplastic agents in breast cancer treatment. **(A)** Forest plot of ROR for CF associated with individual novel antineoplastic agents in the FAERS database. **(B)** Forest plot of ROR for CF associated with individual novel antineoplastic agents in the CANADA database. **(C)** Forest plot of ROR for CF associated with individual novel antineoplastic agents in the JADER database. **(D)** Heatmap illustrating the disproportionality signals (at the PT level) across the three pharmacovigilance databases for various novel antineoplastic agents. Red indicates a statistically significant signal (lower limit of the 95%CI for ROR >1) with at least three reported cases of CF; the intensity of red reflects the magnitude of the ROR. Blue indicates either a non-significant signal (lower limit of 95% CI <1) or fewer than three reported cases. Grey denotes the absence of any CF-related reports. For drugs with case counts between three and five, the results are considered preliminary and unstable, and should be interpreted with caution. ADCs, antibody-drug conjugates; AKT, protein kinase B; CANADA, Canada Vigilance Adverse Reaction Online Database; CDK, cyclin-dependent kinase; CF, cardiac failure; CI, confidence interval; FAERS, Food and Drug Administration’s Adverse Event Reporting System; ICIs, immune checkpoint inhibitors; JADER, Japanese Adverse Drug Event Report; mAbs, monoclonal antibodies; mTOR, mammalian target of rapamycin; NA, not available; PARP, poly adenosine diphosphate-ribose polymerase; PI3K, phosphatidylinositol 3-kinase; PT, preferred term; ROR, reporting odds ratio; TKIs, tyrosine kinase inhibitors.

Disproportionality analyses (**Figures 2A–C**) identified elevated RORs for several agents. In FAERS, trastuzumab showed a strong signal (ROR 2.94, 95% confidence interval [CI] 2.82–3.06), followed by everolimus (ROR 1.59, 95% CI 1.48–1.71) and pertuzumab (ROR 1.78, 95% CI 1.62–1.97). Pertuzumab’s signal was more pronounced in JADER (ROR 6.32, 95% CI 5.60–7.14). Trastuzumab emtansine showed a mild signal in FAERS (ROR 1.30, 95% CI 1.13–1.48), but was non-significant in other databases. In contrast, CDK4/6 inhibitors, PARP inhibitors, and most ICIs consistently showed low or non-significant RORs across all databases. The heatmap (**Figure 2D**) visually summarizes these findings at the PT level, with red, blue, and grey indicating strong, weak, and absent signals, respectively. Trastuzumab consistently appeared as red across all three databases, with high intensity in FAERS and CANADA. Pertuzumab also exhibited red signals in FAERS and JADER, though the signal was less pronounced in CANADA. Everolimus presented as red in FAERS and CANADA, but not in JADER. CDK4/6 inhibitors, ICIs, PARP inhibitors, and PI3K/AKT/mTOR inhibitors were largely blue or grey. Most red signals were limited to HER2-targeted therapies and everolimus. Notably, in accordance with predefined criteria, drugs with fewer than three reported cases were excluded from the disproportionality analysis and their RORs reported as NA. Results for drugs with three to five cases are considered preliminary and unstable and should be interpreted with caution. BCPNN further validated the ROR-based results. Drugs with positive disproportionality, such as trastuzumab and pertuzumab, showed IC_025_ above zero, whereas classes with RORs below 1 showed negative IC_025_. Raw data are shown in **Supplementary Table S3**.

### 3.3 Sensitivity analysis

To validate the ROR method, rosiglitazone (positive control) and tamoxifen (negative control) were analyzed. Rosiglitazone was chosen based on its well-documented association with CF (22). In contrast, tamoxifen was selected for its lack of evidence linking it to CF (23). Rosiglitazone yielded a strong signal (ROR 48.03, 95% CI 10.75–214.62), while tamoxifen showed no association (ROR 0.59, 95% CI 0.48–0.72), supporting the method’s validity.

To account for confounding factors and adjust for reporting bias, adjusted RORs (aRORs) were calculated using multivariate logistic regression models. These analyses confirmed the findings of the unadjusted disproportionality assessment and allowed for a more precise estimation of the relative CF risk associated with each drug. In addition, to minimize the impact of other concomitant drugs that may cause CF, we listed in **Supplementary Table S4** the top 50 drugs most frequently used concomitantly with novel antineoplastic agents in the FAERS database. Based on FDA labelling information, we found that among these drugs, paclitaxel, docetaxel, capecitabine, cyclophosphamide, thyroxine, doxorubicin, epirubicin, and vinorelbine may be associated with the occurrence of CF. We included these eight drugs as confounding variables, along with other relevant variables, in the logistic regression model. In FAERS, trastuzumab (aROR 2.33, 95% CI 2.07–2.63) and everolimus (aROR 1.93, 95% CI 1.62–2.30) remained significantly associated with CF. In contrast, palbociclib (aROR 0.50, 95% CI 0.44–0.58), pembrolizumab (aROR 0.42, 95% CI 0.26–0.70), and atezolizumab (aROR 0.36, 95% CI 0.24–0.54) showed inverse associations. In CANADA, trastuzumab had the highest aROR (8.06, 95% CI 5.96–10.90), while pertuzumab showed a protective association (aROR 0.16, 95% CI 0.04–0.69). In JADER, both trastuzumab (aROR 3.21, 95% CI 1.64–6.27) and pertuzumab (aROR 8.98, 95% CI 5.38–15.00) demonstrated strong associations, consistent with the unadjusted findings (**Table 2**). Additional sensitivity analysis using narrow-scope PTs confirmed these results (**Supplementary Table S5**). Trastuzumab and pertuzumab remained strongly associated with CF across databases, with the highest RORs observed in CANADA and JADER.

**Table 2:** Adjusted ROR for CF associated with novel antineoplastic agents in the treatment of breast cancer.

Drugs	aROR (95% CI)	*P* value
FAERS		
Lapatinib	1.02 (0.83–1.26)	0.83
Tucatinib	0.70 (0.36–1.34)	0.28
Trastuzumab	2.33 (2.07–2.63)	<0.0001
Pertuzumab	1.08 (0.88–1.33)	0.46
Trastuzumab emtansine	0.93 (0.72–1.20)	0.58
Trastuzumab deruxtecan	0.95 (0.73–1.24)	0.72
Sacituzumab govitecan	0.26 (0.13–0.53)	0.0002
Pembrolizumab	0.42 (0.26–0.70)	0.0008
Atezolizumab	0.36 (0.24–0.54)	<0.0001
Palbociclib	0.50 (0.44–0.58)	<0.0001
Ribociclib	0.90 (0.74–1.09)	0.28
Abemaciclib	0.66 (0.47–0.93)	0.0161
Olaparib	0.41 (0.15–1.12)	0.08
Niraparib	1.15 (0.15–8.59)	0.89
Talazoparib	0.60 (0.19–1.90)	0.39
Alpelisib	0.55 (0.35–0.85)	0.0079
Everolimus	1.93 (1.62–2.30)	<0.0001
Capivasertib	1.00 (0.24–4.16)	1.00
CANADA		
Lapatinib	0.79 (0.47–1.32)	0.37
Trastuzumab	8.06 (5.96–10.9)	<0.0001
Pertuzumab	0.16 (0.04–0.69)	0.0142
Trastuzumab emtansine	1.92 (0.62–5.94)	0.26
Trastuzumab deruxtecan	0.88 (0.56–1.36)	0.56
Sacituzumab govitecan	0.71 (0.42–1.19)	0.20
Pembrolizumab	0.24 (0.10–0.60)	0.0021
Palbociclib	0.32 (0.21–0.48)	<0.0001
Ribociclib	0.60 (0.15–2.51)	0.49
Alpelisib	0.92 (0.12–6.96)	0.94
Everolimus	1.25 (0.72–2.15)	0.43
JADER		
Lapatinib	0.39 (0.14–1.11)	0.08
Trastuzumab	3.21 (1.64–6.27)	0.0006
Pertuzumab	8.98 (5.38–15.00)	<0.0001
Trastuzumab emtansine	0.88 (0.41–1.89)	0.75
Trastuzumab deruxtecan	0.46 (0.21–1.01)	0.05
Atezolizumab	0.06 (0.01–0.53)	0.0107
Palbociclib	0.46 (0.20–1.09)	0.08
Olaparib	0.47 (0.11–1.98)	0.30
Everolimus	0.69 (0.27–1.77)	0.44

aROR, adjusted reporting odds ratio; CANADA, Canada Vigilance Adverse Reaction Online Database; CF, cardiac failure; CI, confidence interval; FAERS, FDA Adverse Event Reporting System; JADER, Japanese Adverse Drug Event Report; ROR, reporting odds ratio.

Subgroup analyses restricted to reports from healthcare professionals confirmed the main findings (**Supplementary Figures S1**-**S3**). In FAERS, trastuzumab demonstrated the strongest CF signal (ROR 2.55, 95% CI 2.44–2.67), followed by pertuzumab (ROR 1.60, 95% CI 1.44–1.78) and everolimus (ROR 1.49, 95% CI 1.36–1.62). In CANADA, trastuzumab showed the most prominent signal (ROR 8.79, 95% CI 7.53–10.25). Similar trends were observed in JADER, where trastuzumab (ROR 6.89, 95% CI 5.13–9.25) and pertuzumab (ROR 6.30, 95% CI 5.57–7.13) displayed strong signals, while other agents, including CDK4/6 inhibitors and ICIs, had low or non-significant values. The analysis demonstrated that, in all databases, the drugs signal of disproportionate reporting as reported by healthcare professionals are generally consistent with those identified in the overall reports by all personnel.

### 3.4 Time to onset analysis of CF

**Figure 3:**
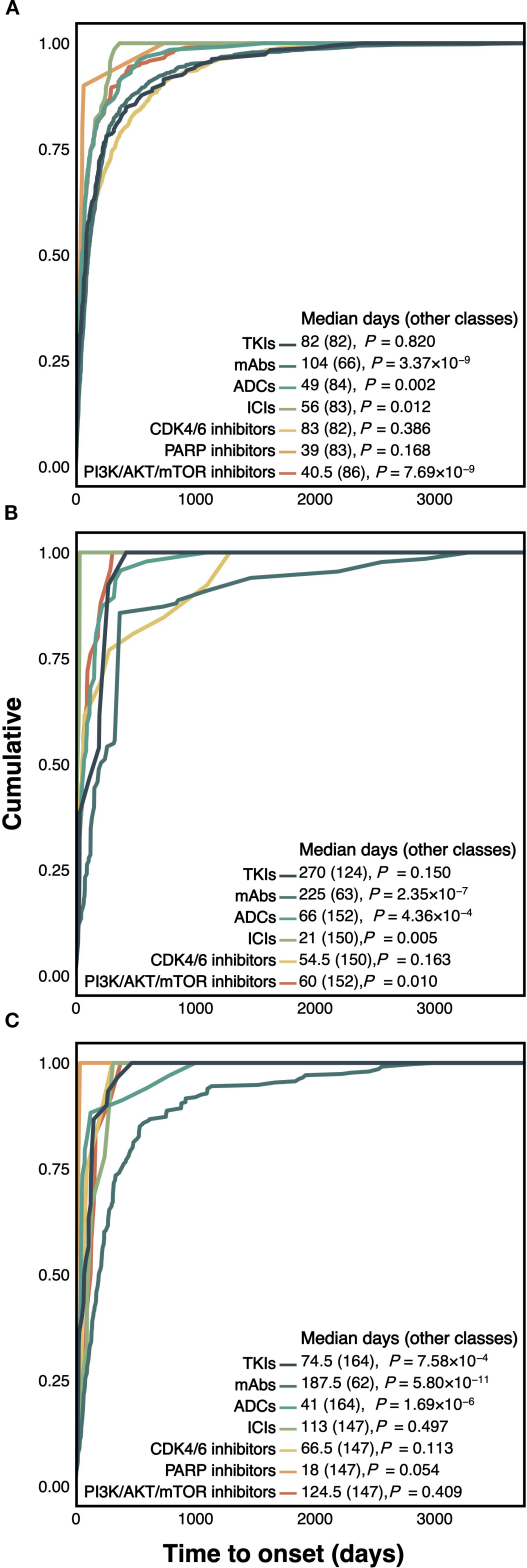
Cumulative distribution curves illustrating the time to onset of treatment-induced CF associated with each novel antineoplastic agent in the FAERS **(A)**, CANADA **(B)**, and JADER **(C)** databases. ADCs, antibody-drug conjugates; AKT, protein kinase B; CANADA, Canada Vigilance Adverse Reaction Online Database; CDK, cyclin-dependent kinase; CF, cardiac failure; FAERS, Food and Drug Administration’s Adverse Event Reporting System; ICIs, immune checkpoint inhibitors; JADER, Japanese Adverse Drug Event Report; mAbs, monoclonal antibodies; mTOR, mammalian target of rapamycin; PARP, poly adenosine diphosphate-ribose polymerase; PI3K, phosphatidylinositol 3-kinase; TKIs, tyrosine kinase inhibitors.

**Figure 3** shows significant variation in median TTO across drug classes. In FAERS, mAbs had the longest median TTO (104 days), significantly longer than other classes (66 days, *P*=3.37×10^-9^). ADCs (49 days), ICIs (56 days), and PI3K/AKT/mTOR inhibitors (40.5 days) had significantly shorter TTOs. Similar trends were observed in CANADA, where TKIs (270 days) and mAbs (225 days) had the longest TTOs, and ADCs (66 days, *P*=4.36×10^-4^) and ICIs (21 days, *P*=0.005) had shorter TTOs. In JADER, mAbs again had the longest TTO (187.5 days, *P*=5.80×10^-11^), whereas ADCs (41 days) and TKIs (74.5 days) showed earlier onset. According to **Supplementary Table S6**, trastuzumab had one of the longest TTOs (FAERS: 105 days; CANADA: 365 days; JADER: 234.5 days), while sacituzumab govitecan had the shortest (FAERS: 10.5 days; CANADA: 6 days). Everolimus showed intermediate or delayed onset depending on database. Weibull analysis indicated that mAbs generally followed an early failure pattern (β < 1). Everolimus and palbociclib showed early failure in FAERS but random failure in CANADA and JADER. Most other agents followed a random failure model.

### 3.5 Analysis of CF cases with fatal outcome

**Figure 4:**
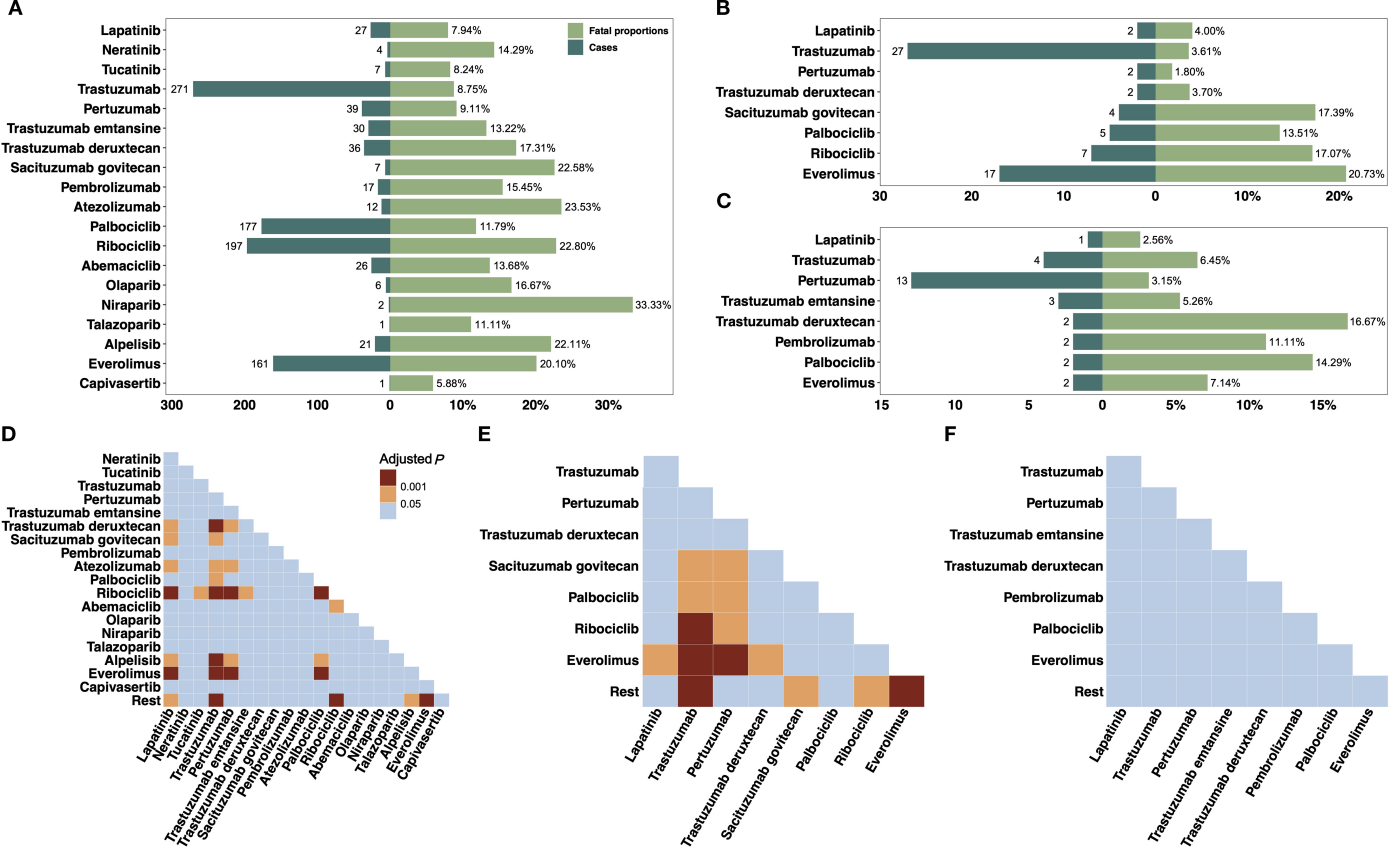
Analysis of CF-associated fatal cases related to novel antineoplastic agents. **(A–C)** display the death proportions and number of cases for individual novel antineoplastic agents in the FAERS, CANADA, and JADER databases, respectively. **(D–F)** present the comparative analysis of CF-related death proportions between each novel antineoplastic agent and all other drugs within the respective databases. Statistical significance was assessed using Pearson chi-square or Fisher’s exact test. CANADA, Canada Vigilance Adverse Reaction Online Database; CF, cardiac failure; FAERS, Food and Drug Administration’s Adverse Event Reporting System; JADER, Japanese Adverse Drug Event Report.

Death proportions among reported CF cases varied by individual agents and databases (**Figures 4A–C**). In FAERS, trastuzumab had the most CF fatal cases (n=271, 8.75%). Niraparib had the highest death proportion (33.3%) but few cases (n=2). Other agents with high fatality included atezolizumab (23.5%), sacituzumab govitecan (22.6%), and everolimus (20.1%). CDK4/6 inhibitors showed moderate rates (abemaciclib 13.7%, palbociclib 11.8%). In CANADA, everolimus again had a high death proportion (20.7%), followed by sacituzumab govitecan (17.4%) and ribociclib (17.1%). Trastuzumab had a lower death proportion (3.6%). In JADER, absolute numbers were smaller. Trastuzumab deruxtecan had the highest death proportion (16.7%), followed by palbociclib (14.3%) and pembrolizumab (11.1%). Statistical comparisons of death proportions between agents were showed in **Figures 4D–F**. In FAERS, death proportions for trastuzumab deruxtecan, ribociclib, and everolimus were significantly higher than those for trastuzumab, pertuzumab, and palbociclib. In CANADA, similar differences were noted, particularly between everolimus and HER2-targeted agents. In JADER, no statistically significant pairwise differences were identified, likely due to limited case numbers per agent.

### 3.6 Comparison with chemotherapy

**Figure 5:**
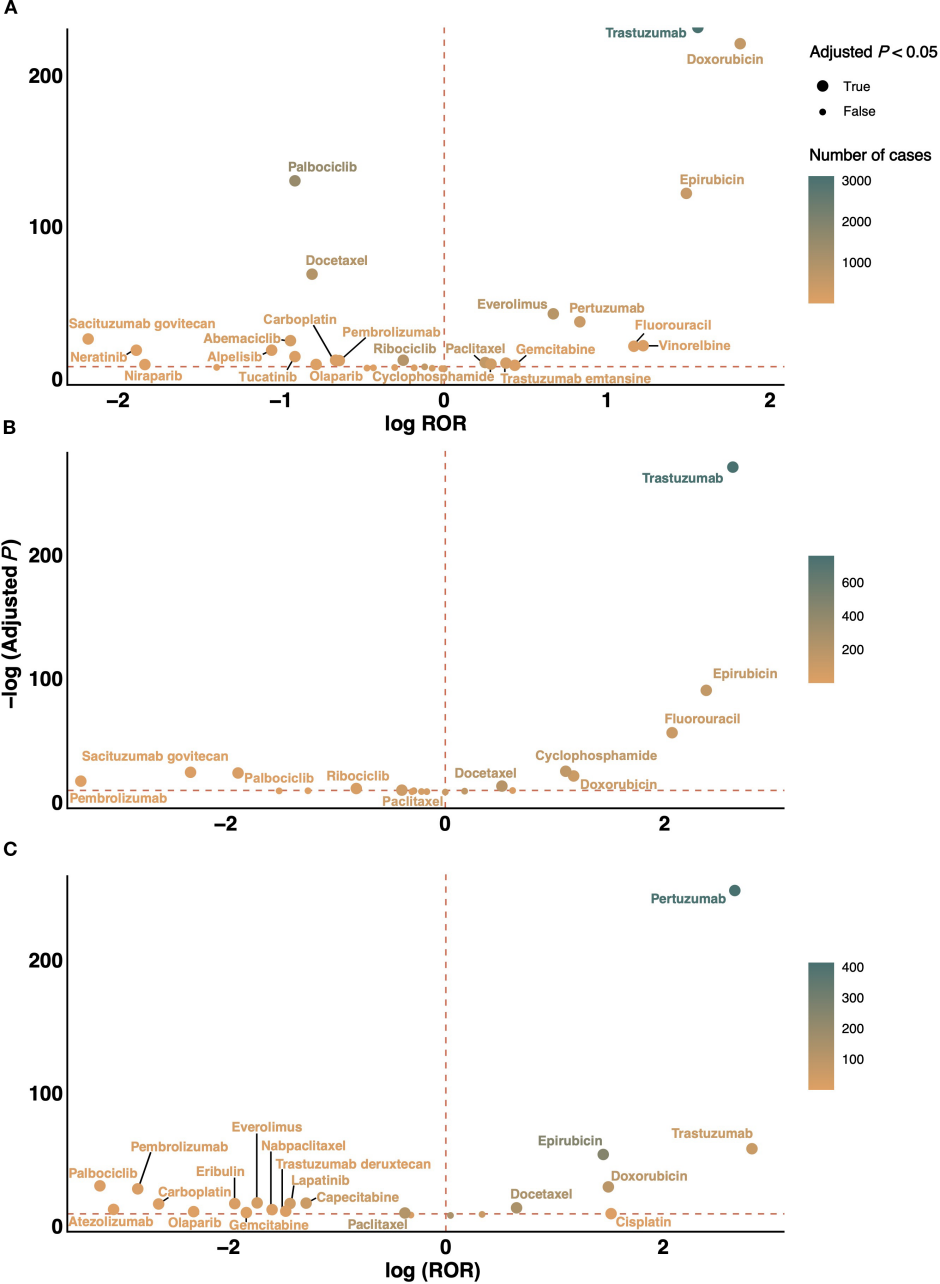
Volcano plots comparing CF signals between novel antineoplastic agents and conventional chemotherapeutic drugs. **(A–C)** depict the comparisons in the FAERS, CANADA, and JADER databases, respectively. The x-axis represents the log-transformed ROR, while the y-axis denotes the negative log10-transformed adjusted P value derived from Fisher’s exact test with Bonferroni correction. Each point represents a specific drug, with color intensity indicating the number of reported CF cases—brighter green corresponding to higher report counts. Drugs located in the upper right quadrant exhibit both elevated CF disproportionality signals and statistically significant associations. CANADA, Canada Vigilance Adverse Reaction Online Database; CF, cardiac failure; FAERS, Food and Drug Administration’s Adverse Event Reporting System; JADER, Japanese Adverse Drug Event Report; ROR, reporting odds ratio.

To contextualize the CF risk of novel agents, we evaluated traditional chemotherapeutic agents, and compared their signals with those of novel agents. In FAERS (**Supplementary Figure S4**), doxorubicin (ROR 3.52) and epirubicin (ROR 2.80) had the highest signals. In CANADA (**Supplementary Figure S5**), epirubicin (ROR 5.20) and fluorouracil (ROR 4.19) ranked highest. In JADER (**Supplementary Figure S6**), doxorubicin (ROR 2.82) and epirubicin (ROR 2.73) again showed strong signals. Volcano plots (**Figure 5**) revealed that trastuzumab and pertuzumab had higher CF signals than chemotherapy. ICIs and CDK4/6 inhibitors clustered in the low-signal region, suggesting lower relative cardiotoxicity.

## 4 Discussion

To our knowledge, this is the first large-scale real-world pharmacovigilance study utilizing data from FAERS, CANADA, and JADER to evaluate CF associated with novel antineoplastic agents used in breast cancer treatment. Our findings indicate that mAbs and mTOR inhibitors are significantly associated with CF. Although no strong signals were observed for ICIs or CDK4/6 inhibitors, potential risk remains and should not be overlooked in clinical practice. These results demonstrate considerable heterogeneity in the risk, timing, and severity of CF across drug classes and agents, offering important insights for clinical decision-making and survivorship care.

Real-world studies, with larger sample sizes, broader demographics, and longer observation periods, provide improved generalizability and a more accurate picture of adverse events in clinical practice. By analyzing three pharmacovigilance databases from different countries, our study incorporated a diverse dataset with a wide range of populations and ethnicities. This cross-national approach enhances the robustness and external validity of our findings. Consistent with previous studies (24), HER2-targeted mAbs, particularly trastuzumab, showed robust and reproducible CF signals in all three databases. Trastuzumab had the highest number of CF reports and the most consistent signal, with elevated RORs and aRORs. These results reinforce its known cardiotoxicity profile (25) and align with clinical guidelines recommending routine cardiac function monitoring during trastuzumab treatment (26). Although pertuzumab was associated with elevated signals in FAERS and JADER, no significant signal was observed in CANADA. Prior studies have reported low rates of symptomatic CF with pertuzumab, and no significant increase in cardiac events when used with other anticancer agents (27, 28). This discrepancy may reflect inter-country differences in reporting practices, drug utilization patterns, or sample size limitations. Additionally, the close co-administration of pertuzumab with trastuzumab may confound attribution in spontaneous reports. These findings underscore the need to interpret disproportionality signals in the context of database characteristics and real-world treatment. Everolimus, an mTOR inhibitor, demonstrated a statistically significant signal for CF in FAERS, but this was not replicated in CANADA or JADER. This inconsistency raises questions about the reliability of the signal. Everolimus has not been strongly associated with CF in pivotal trials such as BOLERO-2 (7), where cardiovascular adverse events were rarely reported. Preclinical studies have even suggested potential cardioprotective effects of mTOR inhibition in certain genetic cardiomyopathy models (29). The positive signal in FAERS may reflect reporting bias, patient complexity, or concomitant medications rather than a direct drug effect. Given the lack of corroboration in other pharmacovigilance systems and the absence of clinical trial evidence, the association between everolimus and CF should be interpreted with caution. Further prospective studies are warranted to clarify whether the observed signal reflects a true pharmacological risk or confounding.

Our analysis consistently demonstrated that CDK4/6 inhibitors, ICIs, and PARP inhibitors were associated with low signals of CF across all three databases, which is notable given their expanding use in both early-stage and metastatic breast cancer. In adjusted analyses, palbociclib was associated with a significantly reduced aROR, in line with recent real-world observational data indicating a low incidence of major adverse cardiovascular events (30). Although ribociclib has been linked to QT prolongation in both clinical trials and real-world cohorts, symptomatic CF remains rare (31). Similarly, ICIs demonstrated no significant association with CF in our analysis and in some analyses were associated with significantly lower odds of reporting. Although myocarditis and pericarditis have emerged as serious but infrequent immune-related adverse events, the incidence of CF remains low in most series (32). PARP inhibitors are primarily associated with hematologic and gastrointestinal toxicity, rather than cardiovascular events. Although isolated reports of hypertension or thromboembolic have been described, there is no established mechanistic link between PARP inhibition and CF (33).

Beyond incidence, understanding the timing of adverse cardiac events is essential for optimizing monitoring strategies and improving early detection. The TTO analysis revealed distinct temporal patterns of CF across drug classes, with important clinical implications. Trastuzumab exhibited the longest median TTO but followed an early failure distribution, suggesting that risk is concentrated shortly after treatment initiation despite delayed median onset. This delayed onset aligns with previous clinical trial showing that trastuzumab-induced cardiotoxicity often manifests after cumulative exposure, especially when used sequentially or concomitantly with anthracyclines (34). In contrast, agents like everolimus showed significantly earlier onset, indicating more acute toxicity profiles. These findings support tailoring cardiac monitoring strategies to drug-specific risk windows—closer surveillance early in treatment for some agents, and extended follow-up for others.

The severity of CF events varied considerably among agents. Everolimus, sacituzumab govitecan, and atezolizumab were associated with the highest death proportions, exceeding 20% in some datasets, whereas trastuzumab demonstrated lower death proportions. Pairwise comparisons in FAERS and CANADA databases confirmed these differences, underscoring the need to evaluate not only incidence but also lethality when assessing cardiovascular risk. However, interpretation of CF-related mortality data requires caution. The attribution of death to CF cannot be definitively confirmed in spontaneous reports, as many cases are confounded by comorbidities, cancer progression, or overall frailty of the oncology population. Additionally, selective reporting of severe or fatal events and heterogeneity in pharmacovigilance systems may distort signal magnitude. Despite these limitations, mortality analysis provides valuable real-world insight into the clinical burden of CF and reinforces the importance of continued post-marketing surveillance for high-risk agents.

Benchmarking against traditional chemotherapeutic agents provides critical context for interpreting the cardiotoxicity of novel treatments. As expected, anthracyclines exhibited the highest CF signals among conventional agents, consistent with their well-documented cumulative dose–dependent cardiotoxicity (35). Notably, the signal strength for agents such as trastuzumab and everolimus was comparable to, or even surpassed, that of anthracyclines in some datasets. This convergence challenges the traditional perception that only cytotoxic chemotherapies carry substantial cardiac risk, reinforcing the need to classify certain targeted agents, particularly HER2-directed therapies, as high-risk from a cardiovascular perspective. Conversely, ICIs and CDK4/6 inhibitors consistently showed lower CF signals than not only novel targeted agents, but also several conventional drugs including fluorouracil and cyclophosphamide. The favorable cardiac safety profiles of CDK4/6 inhibitors and ICIs suggest that newer therapies may offer both oncologic efficacy and cardiovascular sparing, highlighting the importance of integrating cardio-oncology risk assessment into treatment selection and survivorship planning.

Many newer agents have been on the market for a shorter duration compared to established therapies like trastuzumab. Consequently, these newer drugs have less accumulated safety data, which may lead to underreporting of adverse events and, thus, a lower signal detection rate. Additionally, newer agents are often used in combination regimens across various treatment settings (neoadjuvant, adjuvant, and different lines of therapy), which can complicate the attribution of adverse events to a specific drug. These factors, including differential drug exposure times and the complexity of combination therapies, can introduce confounding variables that affect the interpretation of disproportionality analyses. The temporal relationship between drug exposure and adverse event onset is crucial; for instance, adverse events related to cumulative drug exposure may not manifest immediately, potentially leading to underreporting in the early post-marketing phase. Moreover, the concurrent use of multiple agents makes it challenging to establish a clear causal link between a specific drug and an adverse event.

In our study, trastuzumab showed a disproportionality signal for heart failure (ROR=2.94, 95% CI 2.82–3.06) in FAERS, while pertuzumab demonstrated a slightly lower signal (ROR=1.78, 95% CI 1.62–1.97). These findings are consistent with previous evidence indicating that trastuzumab carries the strongest cardiac disorder signals (ROR=3.51), whereas pertuzumab shows a weaker but notable association (ROR=1.98) (18). Although trastuzumab generated the largest number of reports, its mortality proportion was relatively modest, consistent with prior analyses. By contrast, newer agents such as ADCs and TKIs displayed weaker disproportionality signals (ROR < 1.5), likely reflecting their shorter market duration and fewer accumulated reports, in line with prior pharmacovigilance observations that newly approved drugs often show lower signal detection early after launch (15). Importantly, ADCs were linked to higher mortality compared with TKIs, which is in agreement with our findings. Overall, our study confirms previously reported cardiotoxicity signals for established anti-HER2 therapies while providing novel insights into time-to-onset patterns and comparative risk across newer agents, underscoring the complementary role of post-marketing pharmacovigilance in refining cardiac safety profiles.

Strengths of this study include the integration of three complementary pharmacovigilance databases across North America and Asia, allowing for cross-regional validation of cardiac safety signals. We employed robust signal detection methods, including both unadjusted and adjusted models accounting for key confounders, and incorporated positive and negative control drugs to enhance analytical validity. In addition, subgroup analyses restricted to healthcare professional–submitted reports demonstrated high concordance with the overall dataset, reinforcing the reliability of our findings and minimizing concerns of reporting bias.

Despite its strengths, this study has several limitations inherent to spontaneous reporting systems. Underreporting, selective reporting, and absence of denominator data preclude estimation of true incidence rates. Key clinical variables, such as tumor stage, baseline cardiac function, treatment lines, comedications, and cumulative dose were unavailable or incomplete, limiting mechanistic interpretation and leaving residual confounding that may affect the validity of even adjusted analyses. Differences in coding standards, reporting completeness, and drug availability across databases introduce heterogeneity that may affect signal consistency. Moreover, confounding by indication and reporting bias related to drug notoriety cannot be excluded. Although adjusted models were used, causality cannot be inferred from disproportionality signals alone. In addition, findings for drugs with very low case counts, especially those with fewer than five reports, are inherently unstable. Their ROR have wide confidence intervals, making any apparent signal fragile and strictly hypothesis-generating, highlighting the need for caution in interpretation. These limitations highlight the need to integrate pharmacovigilance findings with prospective observational studies and adjudicated clinical data.

In conclusion, our study provides comprehensive real-world evidence characterizing the CF risk of novel antineoplastic agents in breast cancer. While HER2-targeted mAbs and everolimus show elevated and clinically significant cardiotoxicity signals, CDK4/6 inhibitors, PARP inhibitors, and ICIs appear to have more favorable profiles. These results support the need for drug class–specific cardiac surveillance strategies and highlight the value of integrating pharmacovigilance data into treatment planning, particularly when balancing efficacy and safety in long-term breast cancer care.

## Data Availability

The datasets presented in this study can be found in online repositories. The names of the repository/repositories and accession number(s) can be found in the article/**Supplementary Material**.
