# Supplementary material for: Real-world disproportionality analysis of cardiac failure associated with novel antineoplastic agents in breast cancer: a pharmacovigilance study
Source: Front Immunol. 2025 Sep 29;16:1680909. doi: 10.3389/fimmu.2025.1680909 (PMC12515641; doi:10.3389/fimmu.2025.1680909)
Supplement: Supplementary file 1 [file DataSheet1.pdf]

Supplementary materials

1. Statistical analysis

1.1. Reporting odds ratio (ROR)

The ROR and its 95% confidence interval (CI) were calculated using the following formulas:

- **ROR**:

$$ROR=\frac{a/c}{b/d}$$

- **95% CI:**

$$95\%CI=e^{\ln(ROR)\pm1.96\sqrt{\frac{1}{a}+\frac{1}{b}+\frac{1}{c}+\frac{1}{d}}}$$

where:

- a = number of reports of the target event with the target drug
- b = number of reports of non-target events with the target drug
- c = number of reports of the target event with drugs other than the target drug
- d = number of reports of non-target events with drugs other than the target drug

1.2. Bayesian confidence propagation neural network (BCPNN)

To validate the results from ROR, we applied the BCPNN method, which uses the following calculations:

- **IC**:

$$IC=\log_2\frac{a(a+b+c+d)}{(a+b)(a+c)}$$

- **γ**:

$$\gamma=\frac{(a+b+c+d+2)^2}{(a+b+1)(a+c+1)}$$

- **VIC**:

$$VIC=\left(\frac{1}{\ln(2)}\right)^2\left[\frac{a+b+c+d-a+\gamma-2}{(a+2)(1+a+b+c+d+\gamma)}+\frac{a+b+c+d-a-b+1}{(a+b+1)(1+a+b+c+d+2)}+\frac{a+b+c+d-a-c+1}{(a+c+1)(1+a+b+c+d+2)}\right]$$

- **IC_{025}**:

$$IC_{025}=IC-2\sqrt{VIC}$$

This method was selected due to its ability to enhance the stability and accuracy of disproportionality signals, particularly in complex datasets and those with small sample sizes.

2. Supplementary figures

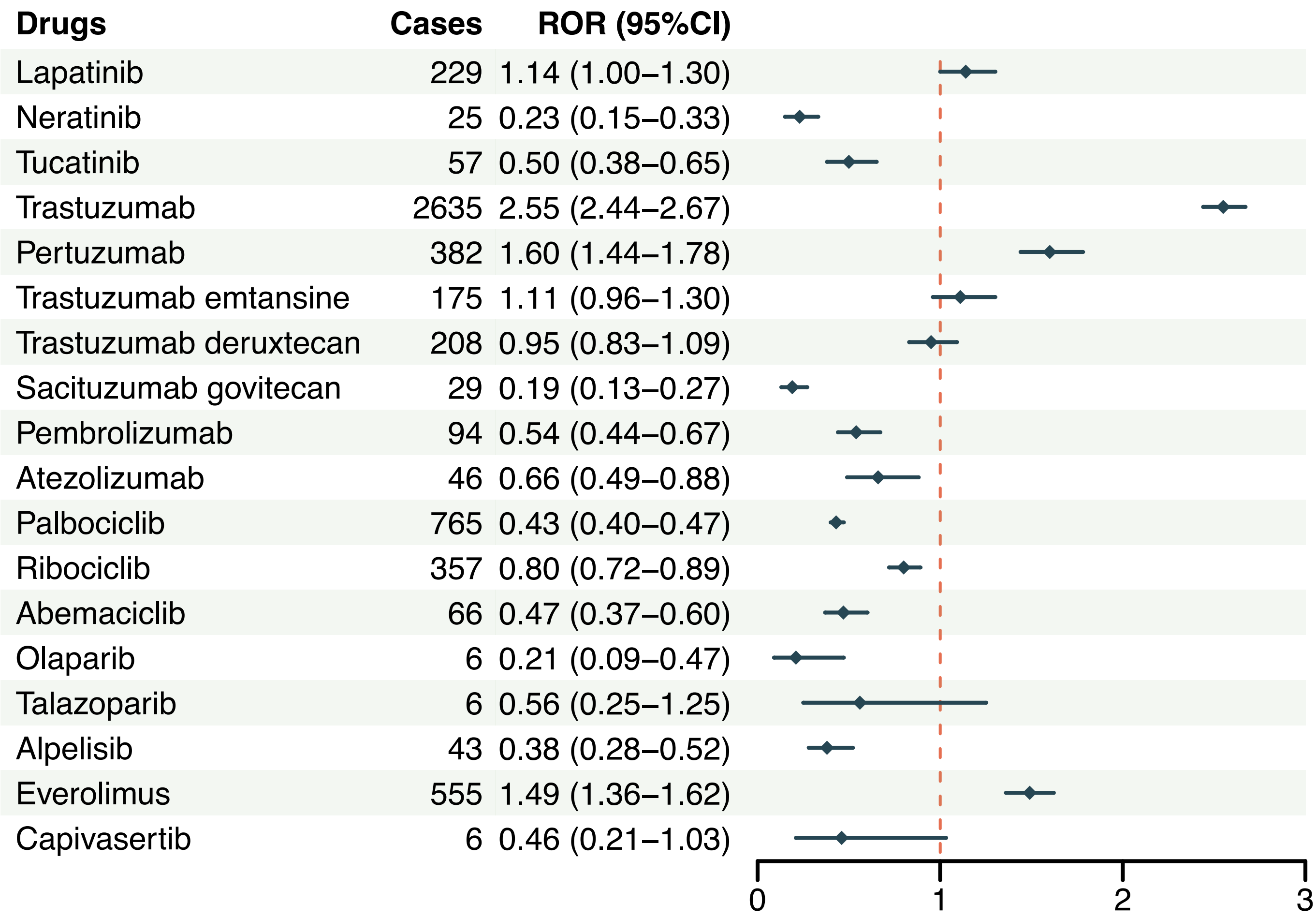

Figure S1. Forest plot of ROR values for different novel antineoplastic agents associated with cardiac failure in FAERS, based on reports from healthcare professionals. Abbreviations: CI, confidence interval; FAERS, FDA Adverse Event Reporting System; ROR, reporting odds ratio.

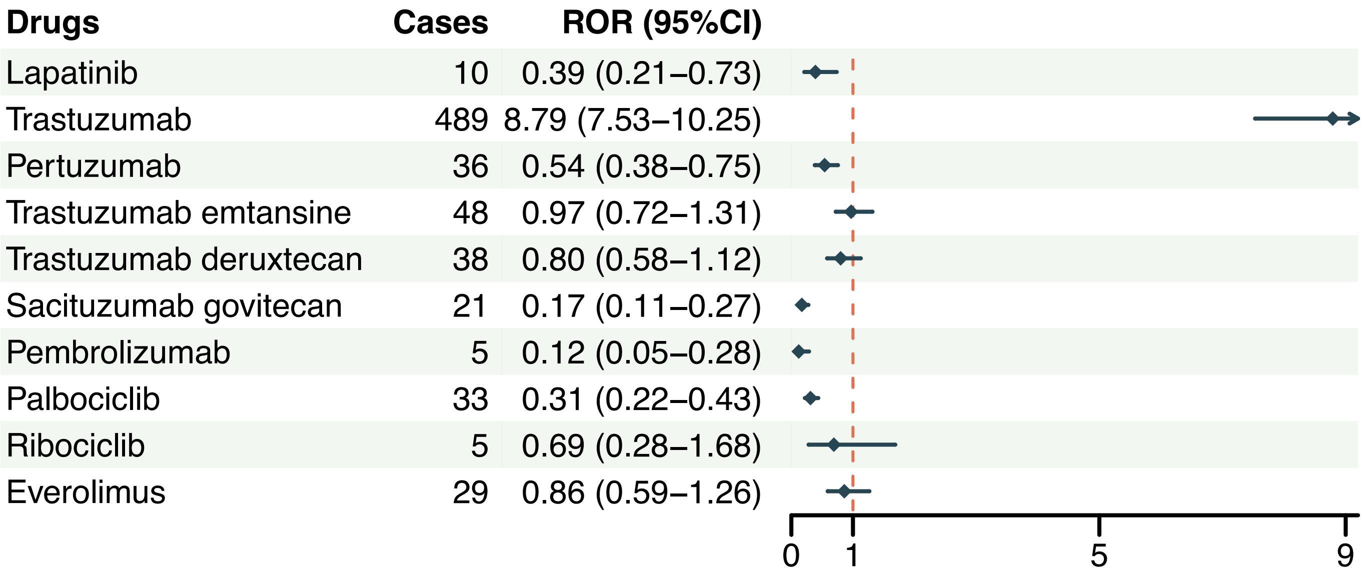

Figure S2. Forest plot of ROR values for different novel antineoplastic agents associated with cardiac failure in CANADA, based on reports from healthcare professionals. Abbreviations: CANADA, Canada Vigilance Adverse Reaction Online Database; CI, confidence interval; ROR, reporting odds ratio.

Drugs	Cases	ROR (95%CI)
Lapatinib	38	0.37 (0.26–0.51)
Trastuzumab	60	6.89 (5.13–9.25)
Pertuzumab	405	6.30 (5.57–7.13)
Trastuzumab emtansine	54	1.20 (0.91–1.58)
Trastuzumab deruxtecan	12	0.37 (0.21–0.66)
Pembrolizumab	17	0.14 (0.08–0.22)
Atezolizumab	3	0.12 (0.04–0.36)
Palbociclib	14	0.11 (0.06–0.18)
Olaparib	4	0.20 (0.08–0.54)
Everolimus	28	0.31 (0.21–0.45)

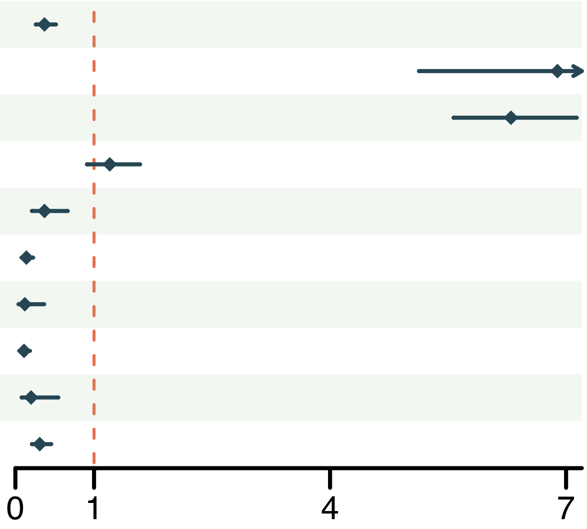

Figure S3. Forest plot of ROR values for different novel antineoplastic agents associated with cardiac failure in JADER, based on reports from healthcare professionals. Abbreviations: CI, confidence interval; JADER, Japanese Adverse Drug Event Report; ROR, reporting odds ratio.

Drugs	Cases	ROR (95%CI)
Paclitaxel	580	1.19 (1.09–1.29)
Nabpaclitaxel	93	0.88 (0.72–1.08)
Docetaxel	915	0.57 (0.53–0.61)
Carboplatin	108	0.63 (0.52–0.76)
Cisplatin	3	0.38 (0.12–1.18)
Doxorubicin	593	3.52 (3.24–3.83)
Epirubicin	491	2.80 (2.55–3.07)
Capecitabine	568	0.92 (0.84–1.00)
Gemcitabine	93	1.35 (1.10–1.66)
Vinorelbine	94	2.33 (1.89–2.86)
Cyclophosphamide	295	1.22 (1.09–1.38)
Eribulin	74	0.99 (0.79–1.25)
Fluorouracil	100	2.24 (1.84–2.74)

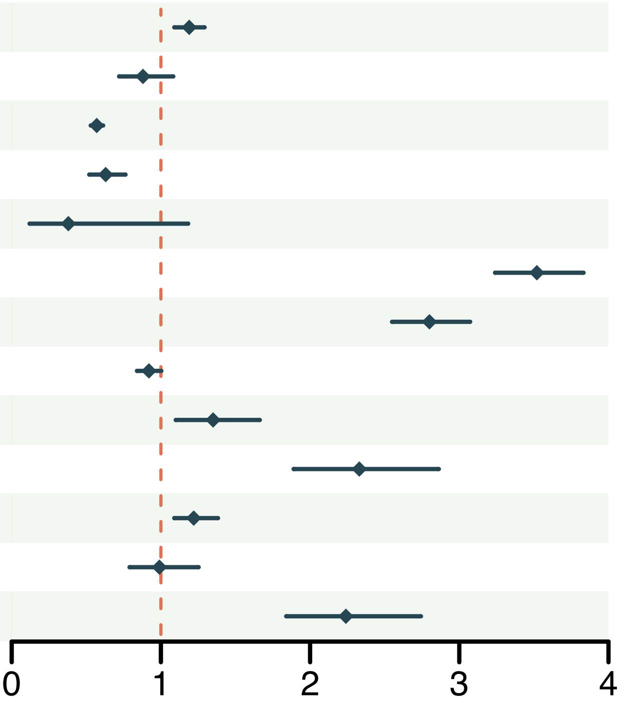

Figure S4. Forest plot of ROR values for different chemotherapeutic agents associated with cardiac failure in FAERS. Abbreviations: ROR, reporting odds ratio; CI, confidence interval; FAERS, FDA Adverse Event Reporting System.

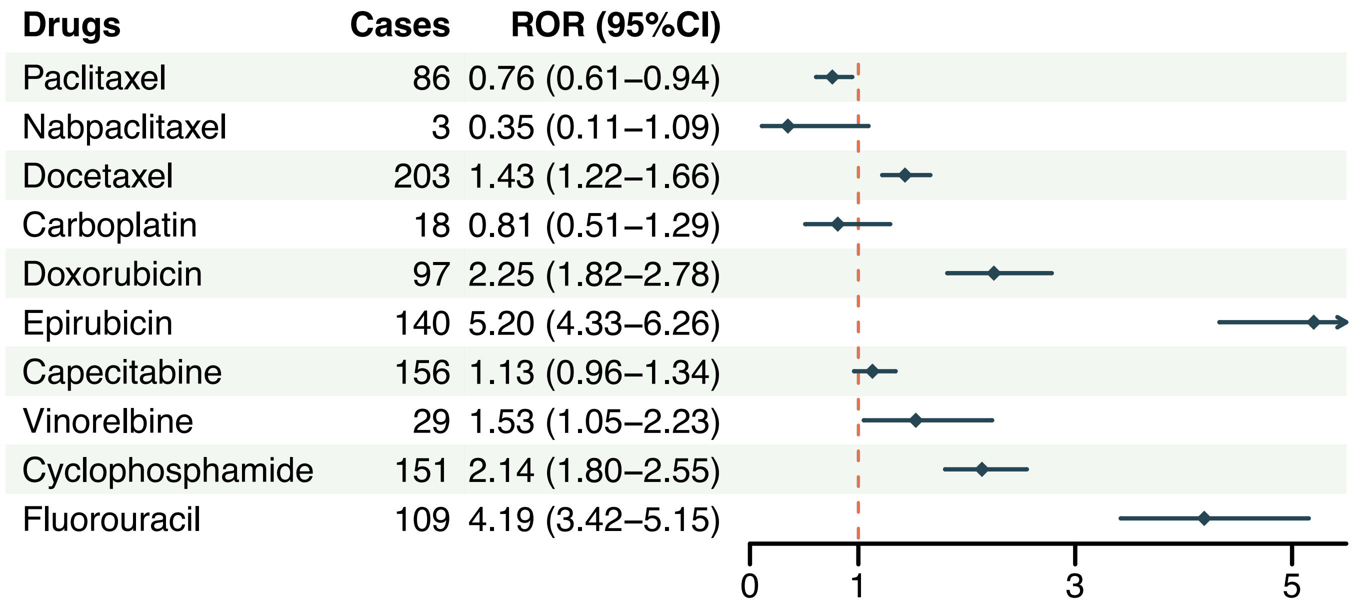

Figure S5. Forest plot of ROR values for different chemotherapeutic agents associated with cardiac failure in CANADA. Abbreviations: ROR, reporting odds ratio; CI, confidence interval; CANADA, Canada Vigilance Adverse Reaction Online Database.

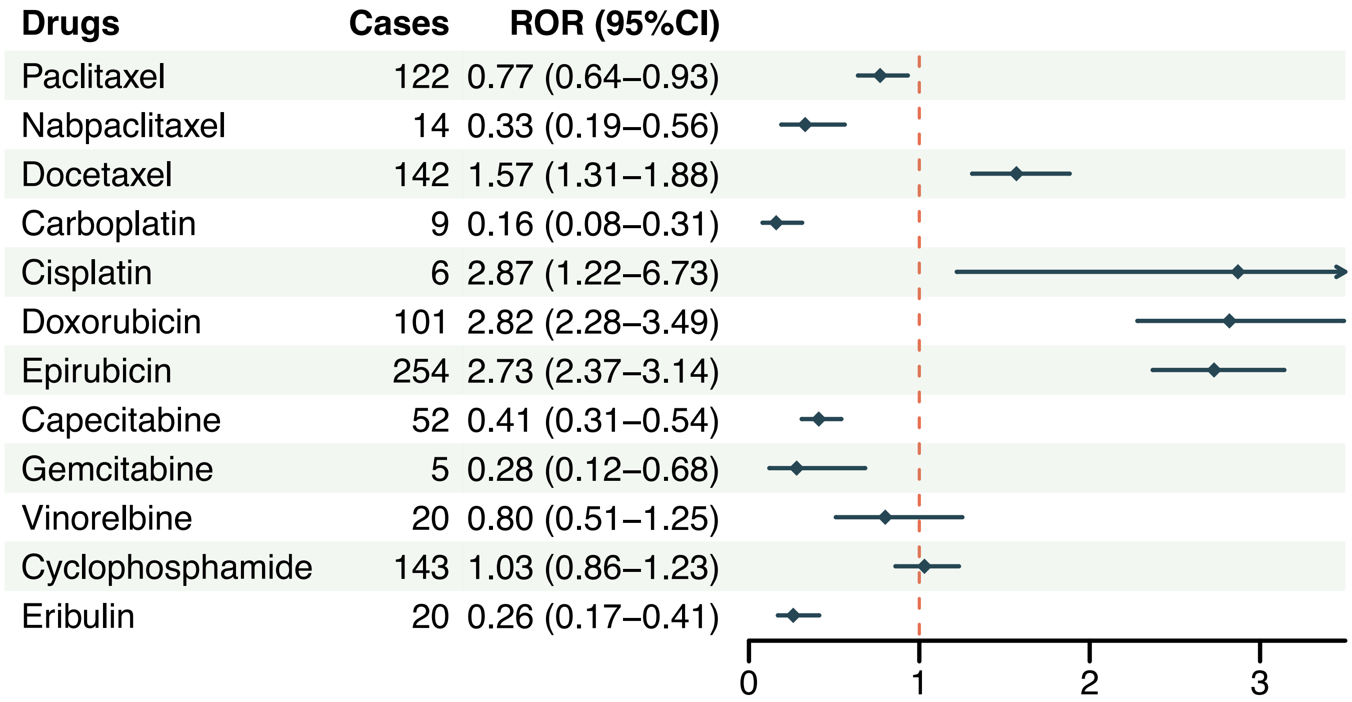

Figure S6. Forest plot of ROR values for different chemotherapeutic agents associated with cardiac failure in JADER. Abbreviations: ROR, reporting odds ratio; CI, confidence interval; JADER, Japanese Adverse Drug Event Report.

3. Supplementary tables

Table S1. List of analyzed drugs

Generic names	Sources	Search terms
Lapatinib	MeSH	• LAPATINIB<br>• N-(3-CHLORO-4-(((3-FLUOROBENZYL)OXY)PHENYL)-6-(5-(((2-METHYLSULFONYL)ETHYL)AMINO)METHYL) -2-FURYL)-4-QUINAZOLINAMINE<br>• GW572016<br>• GW 572016<br>• GW-572016<br>• LAPATINIB DITOSYLATE<br>• TYKERB<br>• GW 282974X<br>• GW-282974X<br>• GW282974X
	JAN	• ラパチニブトシル酸塩水和物
Neratinib	MeSH	• NERATINIB<br>• N-(4-(3-CHLORO-4-(2-PYRIDINYLMETHOXY)ANILINO)-3-CYANO-7-ETHOXY-6-QUINOLYL)-4-(DIMETHYLAMINO)-2-BUTENAMIDE<br>• HKI 272<br>• HKI-272<br>• HKI272<br>• NERLYNX<br>• NERATINIB MALEATE
Tucatinib	MeSH	• TUCATINIB<br>• TUKYSA<br>• IRBINITINIB<br>• ONT-380<br>• N6-(4,4-DIMETHYL-4,5-DIHYDROOXAZOL-2-YL)-N4-(3-METHYL-4-((1,2,4)TRIAZOLO(1,5-A)PYRIDIN-7-YLOXY)PHENYL)QUINAZOLINE-4,6-DIAMINE<br>• N6-(4,5-DIHYDRO-4,4-DMETHYL-2-OXAZOLYL)-N4-(3-METHYL-4-((1,2,4)TRIAZOLO(1,5-A)PYRIDIN-7-YLOXY)PHENYL)-4,6-QUINAZOLINEDIAMINE
Trastuzumab	MeSH	• TRASTUZUMAB<br>• TRASTUZUMAB BETA<br>• BETA, TRASTUZUMAB<br>• HERCEPTIN<br>• TRAZIMERA<br>• TRASTUZUMAB-QYYP<br>• TRASTUZUMAB QYYP
	JAN	• ハーセプチン<br>• トラスツズマブ
Pertuzumab	MeSH	• PERTUZUMAB<br>• OMNITARG<br>• OMNITARG, 2C4<br>• PERJETA<br>• RHUMAB 2C4

Generic names	Sources	Search terms
		RHUMAB-2C4
		RG-1273
	JAN	パージェタ
		ペルツヅマブ
		ペルツズマブ
Trastuzumab emtansine	MeSH	TRASTUZUMAB EMTANSINE
		ADO TRASTUZUMAB EMTANSINE
		TRASTUZUMAB EMTANSINE
		HUN901-DM1
		HUN901DM1
		HUN901 DM1
		KADCYLA
		TRASTUZUMAB-DM1 CONJUGATE
		TRASTUZUMAB DM1 CONJUGATE
		TRASTUZUMAB-DM1
		TRASTUZUMAB DM1
	JAN	トラスツズマブエムタンシン
Trastuzumab deruxtecan	MeSH	TRASTUZUMAB DERUXTECAN
		ENHERTU
		DS-8201A
		DS-8201
		トラスツズマブデルクステカン
Sacituzumab govitecan	MeSH	SACITUZUMAB GOVITECAN
		IMMU-132
		HRS7-SN38
		TRODELVY
Pembrolizumab	MeSH	PEMBROLIZUMAB
		MK-3475
		KEYTRUDA
		LAMBROLIZUMAB
		SCH-900475
	JAN	ペンブロリズマブ
		ペムブロリズマブ
Atezolizumab	MeSH	ATEZOLIZUMAB
		IMMUNOGLOBULIN G1, ANTI-(HUMAN CD ANTIGEN CD274) (HUMAN MONOCLONAL MDPL3280A HEAVY CHAIN), DISULFIDE WITH HUMAN MONOCLONAL MDPL3280A KAPPA-CHAIN, DIMER
		ANTI-PDL1
		MPDL3280A
		MPDL-3280A
		TECENTRIQ
		RG7446
		RG-7446
	JAN	テセントリク
		アテゾリズマブ

Generic names	Sources	Search terms
Palbociclib	MeSH	● PALBOCICLIB
		● 6-ACETYL-8-CYCLOPENTYL-5-METHYL-2-(5-PIPERAZIN-1-YLPYRIDIN-2-YLAMINO)-8H-PYRIDO(2,3-D)PYRIMIDIN-7-ONE
		● PD 0332991
		● PD-0332991
		● PD0332991
		● IBRANCE
	JAN	● パルボシクリブ
Ribociclib	MeSH	● RIBOCICLIB
		● LEE011
		● KISQALI
Abemaciclib	MeSH	● ABEMACICLIB
		● 5-(4-ETHYLPIPERAZIN-1-YLMETHYL)PYRIDIN-2-YL)-(5-FLUORO-4-(7-FLUORO-3-ISOPROPYL-2-METHYL-3H-BENZIMIDAZOL-5-YL)PYRIMIDIN-2-YL)AMINE
		● 5-(4-ETHYLPIPERAZIN-1-YLMETHYL)PYRIDIN-2-YL)-(5-FLUORO-4-(7-FLUORO-3-ISOPROPYL-2-METHYL-3H-BENZOIMIDAZOL-5-YL)PYRIMIDIN-2-YL)AMINE
		● LY2835219
		● LY-2835219
		● LY2385219
		● VERZENIO
		● ABEMACICLIB MESYLATE
		● LY2835210
	JAN	● アベマシクリブ
Olaparib	MeSH	● OLAPARIB
		● AZD 2281
		● AZD-2281
		● AZD2281
		● AZD221
		● LYNPARZA
	JAN	● オラパリブ
Niraparib	MeSH	● NIRAPARIB
		● 2-(4-(PIPERIDIN-3-YL)PHENYL)-2H-INDAZOLE-7-CARBOXAMIDE
		● MK 4827
		● MK-4827
		● MK4827
		● NIRAPARIB HYDROCHLORIDE
		● ZEJULA
	JAN	● ニラパリブトシル酸塩水和物
Talazoparib	MeSH	● TALAZOPARIB
		● BMN 673
		● BMN-673
		● BMN673
		● TALZENNA
Alpelisib	MeSH	● ALPELISIB
		● NVP-BYL719

Generic names	Sources	Search terms
		● BYL719
		● PIQRAY
Everolimus	MeSH	● EVEROLIMUS
		● 40-O-(2-HYDROXYETHYL)-RAPAMYCIN
		● SDZ RAD
		● RAD, SDZ
		● SDZ-RAD
		● RAD 001
		● 001, RAD
		● RAD001
		● CERTICAN
		● AFINITOR
		● ZORTRESS
	JAN	● エベロリムス
		● エベロリムス錠
		● サーティカン
Capivasertib	MeSH	● CAPIVASERTIB
		● 4-AMINO-N-(1-(4-CHLOROPHENYL)-3-HYDROXYPROPYL)-1-(7H-PYRROLO(2,3-D)PYRIMIDIN-4-YL)PIPERIDINE-4-CARBOXAMIDE
		● AZD5363
		● TRUQAP
	JAN	● カピバセルチブ

Abbreviations: JAN, Japanese Accepted Names for Pharmaceuticals; MeSH, Medical Subject Headings.

Table S2. The PTs included in the SMQ of cardiac failure

PT	Code	realm
Acute left ventricular failure	10063081	Narrow
Acute pulmonary oedema	10001029	Narrow
Acute right ventricular failure	10063082	Narrow
Artificial heart implant	10072066	Broad
Atrial natriuretic peptide abnormal	10053410	Broad
Atrial natriuretic peptide increased	10053412	Broad
Bendopnoea	10077819	Broad
Brain natriuretic peptide abnormal	10053408	Broad
Brain natriuretic peptide increased	10053405	Broad
Cardiac asthma	10007522	Narrow
Cardiac cirrhosis	10054936	Broad
Cardiac contractility decreased	10086706	Broad
Cardiac contractility modulation therapy	10077454	Broad
Cardiac device implantation	10088292	Broad
Cardiac device reprogramming	10081886	Broad
Cardiac dysfunction	10079751	Broad
Cardiac failure	10007554	Narrow
Cardiac failure acute	10007556	Narrow
Cardiac failure chronic	10007558	Narrow
Cardiac failure congestive	10007559	Narrow
Cardiac failure high output	10007560	Narrow
Cardiac index decreased	10007577	Broad
Cardiac output decreased	10007595	Broad
Cardiac resynchronisation therapy	10059862	Broad
Cardiac ventriculogram abnormal	10053447	Broad
Cardiac ventriculogram left abnormal	10053499	Broad
Cardiac ventriculogram right abnormal	10053444	Broad
Cardiogenic shock	10007625	Narrow
Cardiohepatic syndrome	10082480	Narrow
Cardiomegaly	10007632	Broad
Cardiopulmonary failure	10051093	Narrow
Cardiorenal syndrome	10068230	Narrow
Cardio-respiratory distress	10049874	Broad
Cardiothoracic ratio increased	10007646	Broad
Central venous pressure increased	10007980	Broad
Chronic left ventricular failure	10063083	Narrow
Chronic myocarditis	10087106	Broad
Chronic right ventricular failure	10063084	Narrow
Congestive hepatopathy	10084058	Narrow
Cor pulmonale	10010968	Narrow
Cor pulmonale acute	10010969	Narrow
Cor pulmonale chronic	10010970	Narrow
Coronary sinus dilatation	10082615	Broad
Diastolic dysfunction	10052337	Broad
Dilatation ventricular	10013012	Broad

PT	Code	realm
Dyspnoea paroxysmal nocturnal	10013974	Broad
Ejection fraction decreased	10050528	Narrow
Global longitudinal strain abnormal	10089038	Broad
Heart and lung transplant	10056409	Broad
Heart failure with midrange ejection fraction	10086366	Narrow
Heart failure with preserved ejection fraction	10076396	Narrow
Heart failure with reduced ejection fraction	10078289	Narrow
Heart transplant	10019314	Broad
Heart transplant failure	10087136	Broad
Hepatic vein dilatation	10069112	Broad
Hepatojugular reflux	10051448	Narrow
Implantable cardiac monitor replacement	10082009	Broad
Intracardiac pressure increased	10079904	Broad
Jugular vein distension	10059865	Broad
Left ventricular diastolic collapse	10080987	Broad
Left ventricular dilatation	10050043	Broad
Left ventricular dysfunction	10049694	Broad
Left ventricular enlargement	10050581	Broad
Left ventricular failure	10024119	Narrow
Low cardiac output syndrome	10024899	Narrow
Lower respiratory tract congestion	10075565	Broad
Myocardial depression	10069140	Broad
Myocardial strain imaging abnormal	10086295	Broad
Neonatal cardiac failure	10049780	Narrow
Neonatal dyspnoea	10084238	Broad
Nocturnal dyspnoea	10049235	Broad
N-terminal prohormone brain natriuretic peptide abnormal	10071660	Broad
N-terminal prohormone brain natriuretic peptide increased	10071662	Broad
Obstructive shock	10073708	Narrow
Oedema	10030095	Broad
Oedema blister	10080039	Broad
Oedema due to cardiac disease	10049632	Broad
Oedema neonatal	10061317	Broad
Oedema peripheral	10030124	Broad
Orthopnoea	10031123	Broad
Peripheral oedema neonatal	10049779	Broad
Peripheral swelling	10048959	Broad
Post cardiac arrest syndrome	10078202	Broad
Prohormone brain natriuretic peptide abnormal	10077783	Broad
Prohormone brain natriuretic peptide increased	10077781	Broad
Pulmonary congestion	10037368	Broad
Pulmonary oedema	10037423	Narrow
Pulmonary oedema neonatal	10050459	Narrow
Right ventricular diastolic collapse	10079613	Broad
Right ventricular dilatation	10074222	Broad
Right ventricular dysfunction	10058597	Broad

PT	Code	realm
Right ventricular ejection fraction decreased	10075337	Narrow
Right ventricular enlargement	10050582	Broad
Right ventricular failure	10039163	Narrow
Scan myocardial perfusion abnormal	10061501	Broad
Stroke volume decreased	10042246	Broad
Systolic dysfunction	10071436	Broad
Venous pressure increased	10047236	Broad
Venous pressure jugular abnormal	10047238	Broad
Venous pressure jugular increased	10047240	Broad
Ventricular assist device insertion	10052371	Broad
Ventricular compliance decreased	10080992	Broad
Ventricular dysfunction	10059056	Broad
Ventricular dyssynchrony	10071186	Broad
Ventricular failure	10060953	Narrow
Ventricular outflow tract dredging	10088350	Broad
Wall motion score index abnormal	10079016	Broad

Abbreviations: PT, preferred term; SMQ, standardized MedDRA query.

Table S3. Raw data used in the disproportionality analysis to calculate the signals

Drugs	a	b	c	d	ROR (95% CI)	IC (IC_{025})
**FAERS**						
Novel antineoplastic agents	8124	489351	8641	583592	**1.12 (1.09–1.16)**	**0.09 (0.05)**
TKIs	453	38434	16312	1034509	0.75 (0.68–0.82)	-0.40 (-0.54)
Lapatinib	340	21749	16425	1051194	1.00 (0.90–1.11)	0.00 (-0.16)
Neratinib	28	6516	16737	1066427	0.27 (0.19–0.40)	-1.85 (-2.38)
Tucatinib	85	10169	16680	1062774	0.53 (0.43–0.66)	-0.89 (-1.21)
mAbs	3525	92388	13240	980555	**2.83 (2.72–2.93)**	**1.26 (1.20)**
Trastuzumab	3097	76856	13668	996087	**2.94 (2.82–3.06)**	**1.33 (1.27)**
Pertuzumab	428	15532	16337	1057411	**1.78 (1.62–1.97)**	**0.80 (0.66)**
ADCs	466	34354	16299	1038589	0.86 (0.79–0.95)	-0.20 (-0.34)
Trastuzumab emtansine	227	11250	16538	1061693	**1.30 (1.13–1.48)**	**0.36 (0.17)**
Trastuzumab deruxtecan	208	13951	16557	1058992	0.95 (0.83–1.09)	-0.07 (-0.27)
Sacituzumab govitecan	31	9153	16734	1063790	0.22 (0.15–0.31)	-2.19 (-2.70)
ICIs	161	15057	16604	1057886	0.68 (0.58–0.80)	-0.54 (-0.77)
Pembrolizumab	110	11002	16655	1061941	0.64 (0.53–0.77)	-0.64 (-0.91)
Atezolizumab	51	4021	16714	1068922	0.81 (0.62–1.07)	-0.30 (-0.70)
CDK4/6 inhibitors	2555	256089	14210	816854	0.57 (0.55–0.60)	-0.64 (-0.70)
Palbociclib	1501	168056	15264	904887	0.53 (0.50–0.56)	-0.80 (-0.88)
Ribociclib	864	64883	15901	1008060	0.84 (0.79–0.90)	-0.23 (-0.33)
Abemaciclib	190	23150	16575	1049793	0.52 (0.45–0.60)	-0.92 (-1.13)
PARP inhibitors	51	6128	16714	1066815	0.53 (0.40–0.70)	-0.90 (-1.30)
Olaparib	36	3974	16729	1068969	0.58 (0.42–0.80)	-0.78 (-1.25)
Niraparib	6	1353	16759	1071590	0.28 (0.13–0.63)	-1.80 (-2.82)
Talazoparib	9	801	16756	1072142	0.72 (0.37–1.39)	-0.47 (-1.35)
PI3K/AKT/mTOR inhibitors	913	46912	15852	1026031	**1.26 (1.18–1.35)**	**0.31 (0.21)**
Alpelisib	95	12662	16670	1060281	0.48 (0.39–0.58)	-1.05 (-1.34)
Everolimus	801	32785	15964	1040158	**1.59 (1.48–1.71)**	**0.63 (0.53)**
Capivasertib	17	1465	16748	1071478	0.74 (0.46–1.20)	-0.42 (-1.09)
**CANADA**						
Novel antineoplastic agents	1049	37168	197	15797	**2.26 (1.94–2.64)**	**0.26 (0.14)**
Lapatinib	53	2593	1193	50372	0.86 (0.65–1.14)	-0.20 (-0.60)
Trastuzumab	756	10629	490	42336	**6.15 (5.47–6.90)**	**1.53 (1.40)**
Pertuzumab	114	5768	1132	47197	0.82 (0.68–1.00)	-0.25 (-0.53)
Trastuzumab emtansine	106	4515	1140	48450	1.00 (0.82–1.22)	0.00 (-0.29)
Trastuzumab deruxtecan	54	2578	1192	50387	0.89 (0.67–1.17)	-0.16 (-0.56)
Sacituzumab govitecan	24	4657	1222	48308	0.20 (0.14–0.31)	-2.16 (-2.74)
Pembrolizumab	5	2026	1241	50939	0.10 (0.04–0.24)	-3.22 (-4.32)
Palbociclib	37	5374	1209	47591	0.27 (0.20–0.38)	-1.75 (-2.22)
Ribociclib	41	2961	1205	50004	0.57 (0.42–0.79)	-0.75 (-1.20)
Alpelisib	5	509	1241	52456	0.42 (0.17–1.00)	-1.24 (-2.34)
Everolimus	86	4389	1160	48576	0.82 (0.66–1.02)	-0.26 (-0.58)
**JADER**						
Novel antineoplastic agents	586	13918	1026	20670	0.85 (0.76–0.94)	-0.14 (-0.28)
Lapatinib	39	2179	1573	32409	0.37 (0.27–0.51)	-1.34 (-1.80)
Trastuzumab	62	195	1550	34393	**7.05 (5.28–9.43)**	**2.44 (2.03)**

Drugs	a	b	c	d	ROR (95% CI)	IC (IC_{025})
Pertuzumab	413	1787	1199	32801	**6.32 (5.60–7.14)**	**2.08 (1.91)**
Trastuzumab emtansine	57	978	1555	33610	1.26 (0.96–1.65)	0.31 (-0.09)
Trastuzumab deruxtecan	12	715	1600	33873	0.36 (0.20–0.63)	-1.43 (-2.21)
Pembrolizumab	18	2548	1594	32040	0.14 (0.09–0.23)	-2.67 (-3.32)
Atezolizumab	3	546	1609	34042	0.12 (0.04–0.36)	-3.03 (-4.32)
Palbociclib	14	2618	1598	31970	0.11 (0.06–0.18)	-3.07 (-3.79)
Olaparib	4	417	1608	34171	0.20 (0.08–0.55)	-2.23 (-3.42)
Everolimus	28	1896	1584	32692	0.30 (0.21–0.44)	-1.61 (-2.15)

Abbreviations: a, reports of target events involving target drugs; ADCs, antibody-drug conjugates; b, reports of non-target events involving target drugs; c, reports of target events involving drugs other than the target drugs; CANADA, Canada Vigilance Adverse Reaction Online Database; CDK4/6, cyclin-dependent kinase 4/6; CI, confidence interval; d, reports of non-target events involving drugs other than the target drugs; FAERS, FDA Adverse Event Reporting System; IC, information component; ICIs, immune checkpoint inhibitors; JADER, Japanese Adverse Drug Event Report; mAbs, monoclonal antibodies; PARP, poly ADP-ribose polymerase; PI3K/AKT/mTOR, phosphatidylinositol 3-kinase/protein kinase B/mammalian target of rapamycin; ROR, reporting odds ratio; TKIs, tyrosine kinase inhibitors.

Table S4. Top 50 concomitant drugs with novel antineoplastic agents in FAERS

Drugs	N	FDA label information
Letrozole	16834	Not common
Fulvestrant	9798	Not common
Paclitaxel	9290	Cardiovascular: congestive heart failure, left ventricular dysfunction, and atrioventricular block. Most patients were previously exposed to cardiotoxic drugs, such as anthracyclines, or had underlying cardiac history
Docetaxel	9139	Cardiovascular: atrial fibrillation, deep vein thrombosis, ECG abnormalities, thrombophlebitis, pulmonary embolism, syncope, tachycardia, myocardial infarction. Ventricular arrhythmia, including ventricular tachycardia, in patients treated with docetaxel in combination regimens including doxorubicin, 5-fluorouracil and/or cyclophosphamide may be associated with fatal outcome<br>BOXED WARNING: Severe fluid retention occurred in 6.5% (6/92) of patients despite use of dexamethasone premedication. It was characterized by one or more of the following events: poorly tolerated peripheral edema, generalized edema, pleural effusion requiring urgent drainage, dyspnea at rest, cardiac tamponade, or pronounced abdominal distention (due to ascites)
Capecitabine	7147	Cardiotoxicity can occur with XELODA. Myocardial infarction/ischemia, angina, dysrhythmias, cardiac arrest, cardiac failure, sudden death, electrocardiographic changes, and cardiomyopathy have been reported with XELODA. These adverse reactions may be more common in patients with a prior history of coronary artery disease
Carboplatin	5608	Not common
Exemestane	4832	Not common
Denosumab	4793	Not common
Vitamin D/Vitamin D3	4343	Not common
Cyclophosphamide	4028	Cardiotoxicity – Myocarditis, myopericarditis, pericardial effusion, arrythmias and congestive heart failure, which may be fatal, have been reported. Monitor patients, especially those with risk factors for cardiotoxicity or pre-existing cardiac disease
Anastrozole	4025	Not common
Dexamethasone	3965	Not common
Ondansetron	3833	Not common
Acetaminophen	3773	Not common
Thyroxine	3740	Cardiovascular: palpitations, tachycardia, arrhythmias, increased pulse and blood pressure, heart failure, angina, myocardial infarction, cardiac arrest
Zoledronic acid	3527	Not common
Omeprazole	2909	Not common
Calcium/Calcium carbonate	2589	Not common
Gabapentin	2453	Not common
Amlodipine	2382	Not common
Pantoprazole	2284	Not common
Metformin	2256	Not common
Atorvastatin	2224	Not common
Furosemide	2213	Not common
Metoprolol	2189	Not common
Tamoxifen	2077	Not common
Aspirin	2073	Not common

Drugs	N	FDA label information
Doxorubicin	2061	BOXED WARNING: Cardiomyopathy: Myocardial damage can occur with doxorubicin hydrochloride with incidences from 1% - 20% for cumulative doses from 300 mg/m2 to 500 mg/m2 when doxorubicin hydrochloride is administered every 3 weeks. The risk of cardiomyopathy is further increased with concomitant cardiotoxic therapy. Assess left ventricular ejection fraction (LVEF) before and regularly during and after treatment with doxorubicin hydrochloride
Loperamide	2017	Not common
Oxycodone	1978	Not common
Lorazepam	1945	Not common
Epirubicin	1931	BOXED WARNING: Cardiac Toxicity: Myocardial damage, including acute left ventricular failure, can occur with ELLENCE. The risk of cardiomyopathy is proportional to the cumulative exposure with incidence rates from 0.9% at a cumulative dose of 550 mg/m2, 1.6% at 700 mg/m2, and 3.3% at 900 mg/m2. The risk of cardiomyopathy is further increased with concomitant cardiotoxic therapy. Assess left ventricular ejection fraction (LVEF) before and regularly during and after treatment with ELLENCE (5.1)
Lisinopril	1849	Not common
Ibuprofen	1721	Not common
Vinorelbine	1680	Cardiovascular toxicity: Chest pain occurred in 5% of patients
Losartan	1536	Not common
Alprazolam	1499	Not common
Tramadol	1421	Not common
Sodium chloride	1409	Not common
Morphine	1406	Not common
Prochlorperazine	1369	Not common
Vitamin B12	1263	Not common
Ascorbic acid	1234	Not common
Metoclopramide	1206	Not common
Simvastatin	1172	Not common
Pregabalin	1131	Not common
Goserelin	1111	Not common
Apixaban	1099	Not common
Hydrochlorothiazide	1050	Not common
Bisoprolol	1010	Not common

Abbreviations: FAERS, FDA Adverse Event Reporting System.

Table S5. ROR for novel antineoplastic agents in breast cancer using PTs of the narrow SMQ in cardiac failure

Drugs	FAERS		CANADA		JADER	
	Cases	ROR (95%CI)	Cases	ROR (95%CI)	Cases	ROR (95%CI)
Lapatinib	165	1.01 (0.87–1.18)	18	0.48 (0.30–0.77)	23	0.28 (0.19–0.43)
Neratinib	10	0.21 (0.11–0.38)	NA	NA	NA	NA
Tucatinib	26	0.34 (0.23–0.50)	NA	NA	NA	NA
Trastuzumab	1959	4.16 (3.95–4.38)	571	13.41 (11.27–15.94)	43	5.85 (4.20–8.16)
Pertuzumab	254	2.22 (1.95–2.51)	56	0.67 (0.51–0.88)	310	5.86 (5.11–6.72)
Trastuzumab emtansine	144	1.72 (1.46–2.04)	45	0.69 (0.51–0.94)	41	1.17 (0.85–1.61)
Trastuzumab deruxtecan	122	1.17 (0.98–1.40)	33	0.91 (0.64–1.30)	9	0.35 (0.18–0.68)
Sacituzumab govitecan	14	0.20 (0.12–0.35)	NA	NA	NA	NA
Pembrolizumab	52	0.63 (0.48–0.83)	NA	NA	14	0.15 (0.09–0.25)
Atezolizumab	33	1.10 (0.78–1.55)	NA	NA	3	0.15 (0.05–0.48)
Palbociclib	530	0.38 (0.35–0.42)	21	0.26 (0.17–0.40)	5	0.05 (0.02–0.12)
Ribociclib	305	0.61 (0.55–0.69)	14	0.33 (0.19–0.55)	NA	NA
Abemaciclib	77	0.44 (0.35–0.55)	NA	NA	NA	NA
Olaparib	19	0.64 (0.41–1.01)	NA	NA	3	0.20 (0.06–0.63)
Niraparib	3	0.30 (0.10–0.92)	NA	NA	NA	NA
Talazoparib	6	1.01 (0.45–2.24)	NA	NA	NA	NA
Alpelisib	28	0.29 (0.20–0.43)	NA	NA	NA	NA
Everolimus	280	1.14 (1.01–1.28)	19	0.29 (0.18–0.46)	19	0.27 (0.17–0.43)
Capivasertib	10	0.91 (0.49–1.70)	NA	NA	NA	NA

Abbreviations: CANADA, Canada Vigilance Adverse Reaction Online Database; CI, confidence interval; FAERS, FDA Adverse Event Reporting System; JADER, Japanese Adverse Drug Event Report; PT, preferred term; ROR, reporting odds ratio; SMQ, standardized MedDRA query.

Table S6. TTO and Weibull shape parameters for cardiac failure associated with novel antineoplastic agents

Drugs	TTO (days): median (IQR)	Scale parameter: alpha (95% CI)	Shape parameter: beta (95% CI)	Type
**FAERS**				
Lapatinib	83 (22–222)	165.14 (122.19–208.10)	0.62 (0.55–0.69)	Early failure
Neratinib	14 (3.5–28.25)	20.43 (2.36–38.49)	0.83 (0.38–1.28)	Random failure
Tucatinib	183 (47–492)	275.63 (106.51–444.76)	0.93 (0.51–1.35)	Random failure
Trastuzumab	105 (42–235.5)	195.13 (174.68–215.59)	0.74 (0.70–0.78)	Early failure
Pertuzumab	91 (39–232)	167.61 (122.87–212.36)	0.70 (0.61–0.79)	Early failure
Trastuzumab emtansine	43 (22–118)	108.04 (64.37–151.71)	0.64 (0.52–0.75)	Early failure
Trastuzumab deruxtecan	72.5 (25.5–149.75)	114.74 (77.74–151.74)	0.89 (0.70–1.08)	Random failure
Sacituzumab govitecan	10.5 (7–21.5)	22.55 (7.67–37.42)	0.91 (0.54–1.29)	Random failure
Pembrolizumab	58.5 (23–107.25)	79.91 (51.66–108.16)	1.00 (0.73–1.27)	Random failure
Atezolizumab	53 (12–148)	89.70 (48.65–130.75)	0.79 (0.57–1.00)	Random failure
Palbociclib	133 (33.75–445.25)	245.52 (205.70–285.35)	0.71 (0.65–0.76)	Early failure
Ribociclib	42 (13–225.5)	122.70 (98.53–146.87)	0.59 (0.54–0.64)	Early failure
Abemaciclib	101.5 (26–265.5)	165.53 (103.39–227.66)	0.71 (0.58–0.85)	Early failure
Olaparib	22 (19–42)	36.35 (18.57–54.12)	1.90 (0.62–3.18)	Random failure
Alpelisib	13 (8–54.5)	48.04 (18.49–77.58)	0.61 (0.46–0.76)	Early failure
Everolimus	47 (18–142)	101.13 (82.48–119.78)	0.71 (0.64–0.77)	Early failure
**CANADA**				
Lapatinib	270 (30–419)	614.75 (58.05–1171.46)	0.56 (0.36–0.76)	Early failure
Trastuzumab	365 (122–365)	480.96 (359.59–602.33)	0.82 (0.70–0.94)	Early failure
Pertuzumab	115 (1–181.5)	92.41 (28.71–156.11)	0.51 (0.37–0.65)	Early failure
Trastuzumab emtansine	117 (63–217)	182.52 (91.50–273.54)	0.90 (0.62–1.19)	Random failure
Trastuzumab deruxtecan	90 (49–158)	151.58 (85.41–217.74)	1.32 (0.76–1.87)	Random failure
Sacituzumab govitecan	6 (1–7)	8.12 (1.22–15.02)	0.68 (0.41–0.95)	Early failure
Palbociclib	145 (16.5–666.25)	261.39 (82.74–668.81)	0.60 (0.42–1.23)	Random failure
Ribociclib	53.5 (18–155.75)	139.04 (21.62–256.47)	0.62 (0.40–0.84)	Early failure
Everolimus	60 (28–120)	98.95 (61.09–136.81)	1.08 (0.76–1.41)	Random failure
**JADER**				
Lapatinib	74.5 (20.25–128.75)	102.39 (63.25–141.52)	0.99 (0.72–1.26)	Random failure
Trastuzumab	234.5 (106.25–504.25)	434.72 (350.38–519.05)	0.83 (0.74–0.92)	Early failure
Pertuzumab	175 (75.5–307.75)	260.13 (218.41–301.86)	0.99 (0.88–1.10)	Random failure
Trastuzumab emtansine	41 (21–53)	108.35 (45.26–171.43)	0.72 (0.52–0.91)	Early failure
Trastuzumab deruxtecan	38 (21–77.5)	56.72 (30.91–82.54)	1.61 (0.73–2.49)	Random failure
Pembrolizumab	102.5 (76.5–157)	133.15 (61.49–204.81)	1.35 (0.58–2.12)	Random failure
Palbociclib	61 (45–137.5)	104.27 (22.36–186.17)	0.99 (0.41–1.58)	Random failure
Everolimus	124.5 (75.5–157.75)	153.55 (90.47–216.64)	1.45 (0.81–2.08)	Random failure

Abbreviations: CANADA, Canada Vigilance Adverse Reaction Online Database; CI, confidence interval; FAERS, FDA Adverse Event Reporting System; IQR, interquartile range; JADER, Japanese Adverse Drug Event Report; TTO, time to onset.
